# Renal glycosuria triggers a coordinated mannose and glutamine metabolic program to maintain glucose homeostasis

**DOI:** 10.1016/j.celrep.2026.117804

**Published:** 2026-08-12

**Authors:** Nadia Rashid, Moses Otunla, Nazmul Hasan, Michael J. Hodges, Heba H. Qaissi, Tumininu S. Faniyan, Pontiana Ritika Clement, Penghui Lin, Mohamed M.Y. Kaddah, Teresa A. Cassel, Donald A. Morgan, Kamal Rahmouni, Kavaljit H. Chhabra

**Affiliations:** 1 Department of Pharmacology and Nutritional Sciences, University of Kentucky, Lexington, KY, USA; 2 Division of Endocrinology and Metabolism, University of Rochester, Rochester, NY, USA; 3 Center for Environmental and Systems Biochemistry, University of Kentucky, Lexington, KY, USA; 4 Markey Cancer Center, University of Kentucky, Lexington, KY, USA; 5 Department of Neuroscience and Pharmacology, University of Iowa Carver College of Medicine, Iowa City, IA, USA; 6 Veterans Affairs Health Care System, Iowa City, IA, USA; 7 These authors contributed equally; 8 Lead contact

## Abstract

Glycosuria, whether genetically induced or triggered by SGLT2 inhibitors, activates compensatory glucose-producing pathways that limit glucose lowering in type 2 diabetes. To define these pathways, we studied renal *Glut2* knockout mice, which progressively lose *Slc5a2* (encoding SGLT2) expression yet maintain normoglycemia despite marked urinary glucose loss. Metabolic profiling and isotope tracing revealed coordinated adaptations in mannose and glutamine metabolism during glycosuria. Skeletal muscle reduced glucose utilization and showed increased incorporation of mannose-derived carbon into oxidative metabolism while whole-body glycolysis declined, establishing a systemic glucose-sparing state. Disruption of glutamine transport or mannose utilization caused hypoglycemia in mice treated with an SGLT2 inhibitor, consistent with reliance on these substrates to maintain glucose homeostasis during glycosuria. Multiomic profiling revealed increased expression and chromatin accessibility of mannose and glutamine transport pathways. These findings identify a kidney-coordinated metabolic program associated with maintenance of systemic glucose homeostasis during glycosuria and may inform strategies to optimize the glucose-lowering efficacy of SGLT2 inhibitors.

## In brief

Rashid et al. show that during renal glycosuria, the kidney coordinates a mannose and glutamine metabolic program that sustains systemic glucose homeostasis. Skeletal muscle spares glucose while mannose- and glutamine-derived carbon supports glucose production, revealing adaptations that may inform strategies to improve the glucose-lowering efficacy of SGLT2 inhibitors.

## Graphical Abstract

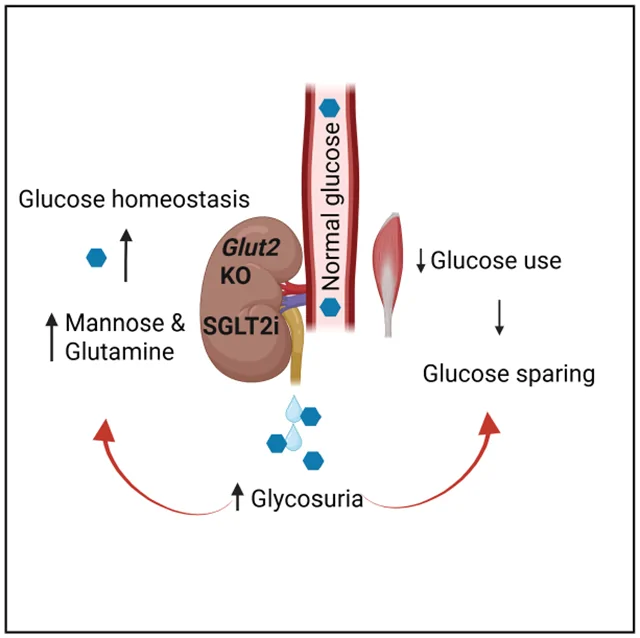

## INTRODUCTION

The kidney regulates systemic glucose homeostasis by reabsorbing filtered glucose through transporters such as GLUT2 and SGLT2. When this process is disrupted, large amounts of glucose are lost in the urine (renal glycosuria); yet, blood glucose levels often remain remarkably stable—a paradox that highlights unknown compensatory mechanisms. Genetic deficiency of *GLUT2*^1,2^ or *SGLT2*^3^ causes renal glycosuria, resulting in high urinary glucose excretion despite normal blood glucose. Similarly, SGLT2 inhibitors increase glucose excretion in urine and are used to manage type 2 diabetes.^4^ However, their glucose lowering efficacy is attenuated^5^ in part due to endogenous glucose compensation that offsets the glucose loss in urine.

Consistent with this concept, healthy individuals^6^ and rodents^7^ treated with SGLT2 inhibitors maintain normal blood glucose levels despite massive glycosuria. A similar phenotype is observed in mice lacking renal *Glut2*^2^ or *Sglt2*,^8^ which maintain normoglycemia through compensatory glucose production. Previous studies suggest that central mechanisms contribute to this response: the hypothalamus regulates glucose reabsorption^9-11^ through renal GLUT2 and promotes glucose production^12^ during glycosuria. Renal nerves have also been implicated in this compensatory response, although their contribution appears to be partial and context dependent.^12-14^ For example, renal denervation reduces compensatory glucose production by only ~50% in renal *Glut2* knockout (r*Glut2* KO) mice,^12^ indicating that additional mechanisms are responsible for glucose compensation. Similarly, renal denervation in human subjects treated with SGLT2 inhibitors does not affect compensatory glucose production.^13^ By contrast, glucose production in response to SGLT2 inhibitors is attenuated in another study involving kidney transplantation and bilateral nephrectomy.^14^ Thus, the metabolic pathways that sustain glucose homeostasis during chronic renal glycosuria remain undefined, and identifying these adaptations may reveal strategies to enhance the glucose-lowering efficacy of SGLT2 inhibitors in type 2 diabetes.

To investigate systemic adaptations to chronic renal glucose loss, we studied mouse models of renal glycosuria. These include r*Glut2* KO mice,^2^ which develop progressive loss of *Slc5a2* expression (encoding SGLT2) and marked urinary glucose excretion while maintaining normal blood glucose levels. This phenotype has been independently confirmed and reviewed by other research groups,^15,16^ validating r*Glut2* KO mice as a genetic model of sustained renal glycosuria. We also examined mice treated with the SGLT2 inhibitor dapagliflozin to assess whether similar metabolic adaptations occur during pharmacologically induced glycosuria.

Here, we identify coordinated regulation of mannose and glutamine metabolism as key components of a kidney-driven metabolic program that maintains systemic glucose homeostasis during persistent glycosuria.

## RESULTS

### Renal glycosuria induces metabolic rewiring in the kidney

**Figure 1.**
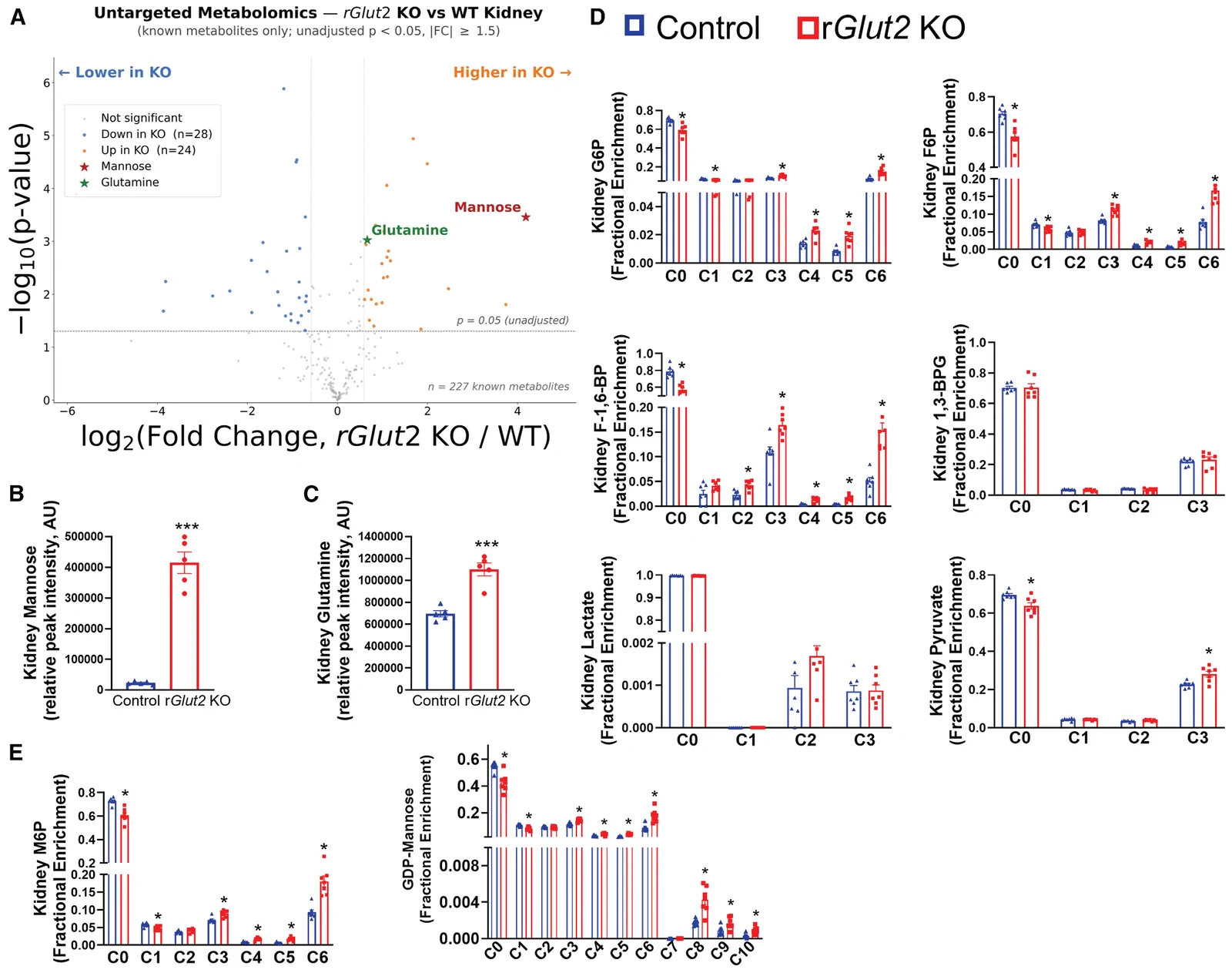
Untargeted metabolomics and stable isotope-resolved metabolomics of kidney glucose and mannose metabolism in 14-week-old male r*Glut2* KO mice and their control littermates 60 min after ^13^C_6_-glucose administration (A) Volcano plot of untargeted metabolomics data comparing r*Glut2* KO vs. wild-type kidneys (known metabolites only; unadjusted *p* < 0.05, ∣FC∣ ≥ 1.5). Blue dots indicate metabolites lower in KO (*n* = 28); orange dots indicate metabolites higher in KO (*n* = 24). Mannose and glutamine are highlighted. (B) Kidney mannose abundance in r*Glut2* KO mice and controls (relative peak intensity, AU). (C) Kidney glutamine abundance in r*Glut2* KO mice and controls (relative peak intensity, AU). (D) ^13^C-glucose fractional enrichment of glycolytic intermediates G6P, F6P, fructose 1,6-bisphosphate (F-1,6-BP), 1,3-BPG, lactate, and pyruvate in r*Glut2* KO kidneys relative to controls, consistent with altered glycolytic labeling patterns. (E) ^13^C_6_-glucose fractional enrichment of mannose 6-phosphate (M6P) and GDP-mannose was increased in r*Glut2* KO kidneys, indicating enhanced routing of hexose carbons into the mannose/M6P pathway during glucose loss. Fractional enrichment values represent the fraction of each metabolite derived from ^13^C_6_-glucose, corrected for natural isotope abundance. *N* = 7 (D and E), ***p < 0.001, *p < 0.05, multiple comparison unpaired *t* test corrected using Holm-Sidak method (D and E); two-tailed unpaired *t* test (B and C). Data in (B–E) are represented as mean ± SEM.

To understand how systemic glucose homeostasis is maintained during chronic renal glycosuria, we examined metabolic adaptations in the kidney using untargeted metabolomics in r*Glut2* KO mice. We observed that mannose levels were about 18-fold higher in the KO mice compared with their control littermates (Figures 1A and 1B and line 99, Data S1). The KO mice also had high levels of glutamine (Figure 1C), tyrosine and glycine (lines 145, 25, and 140, respectively, Data S1), suggesting altered amino acid handling. In contrast, monosaccharides such as galactose and fructose were decreased (lines 153 and 159, respectively, Data S1) in the KO mice. This metabolic profile resembles adaptations observed during glucose deprivation and suggests that alternative carbon sources, including mannose and glutamine, may contribute to maintaining glucose homeostasis during renal glycosuria. We focused on examining these two metabolites for subsequent analyses because mannose has been suggested to support energy homeostasis during high metabolic demands^17-19^ and glutamine is a major substrate for gluconeogenesis in the kidney.^20-22^

To comprehensively investigate the role of glucose and mannose metabolism in energy homeostasis during renal glycosuria, we used a stable isotope tracing technique. We administered ^13^C_6_ glucose (oral gavage, 60 mg/mouse) to mice and tracked the incorporation of ^13^C into downstream metabolites 60 min after the labeled glucose administration. In the kidneys of r*Glut2* KO mice, the ^13^C incorporation was increased in proximal glycolytic intermediates, including glucose-6-phosphate (G6P) and fructose-6-phosphate (F6P), as well as in downstream metabolites such as pyruvate and lactate (Figure 1D; quantitative metabolite abundances in μmol/g protein are shown in Figure S4A). At the same time, fractional enrichment of the intermediate metabolite 1,3-bisphosphoglycerate (1,3-BPG) showed no proportional increase relative to upstream intermediates. This discontinuity is consistent with reversible reactions in upper glycolysis and redistribution of carbon through glucose cycling within the pathway. ^13^C-glucose fractional enrichment into mannose metabolites was increased in the kidney (Figure 1E; quantitative data in Figure S4B), suggesting that during renal glycosuria, glucose-derived carbon is routed into the mannose pathway via mannose-6-phosphate (M6P) isomerase (MPI), as illustrated in Figure S1. This is consistent with increased routing of hexose carbons through the mannose pathway during glucose loss.

**Figure 2.**
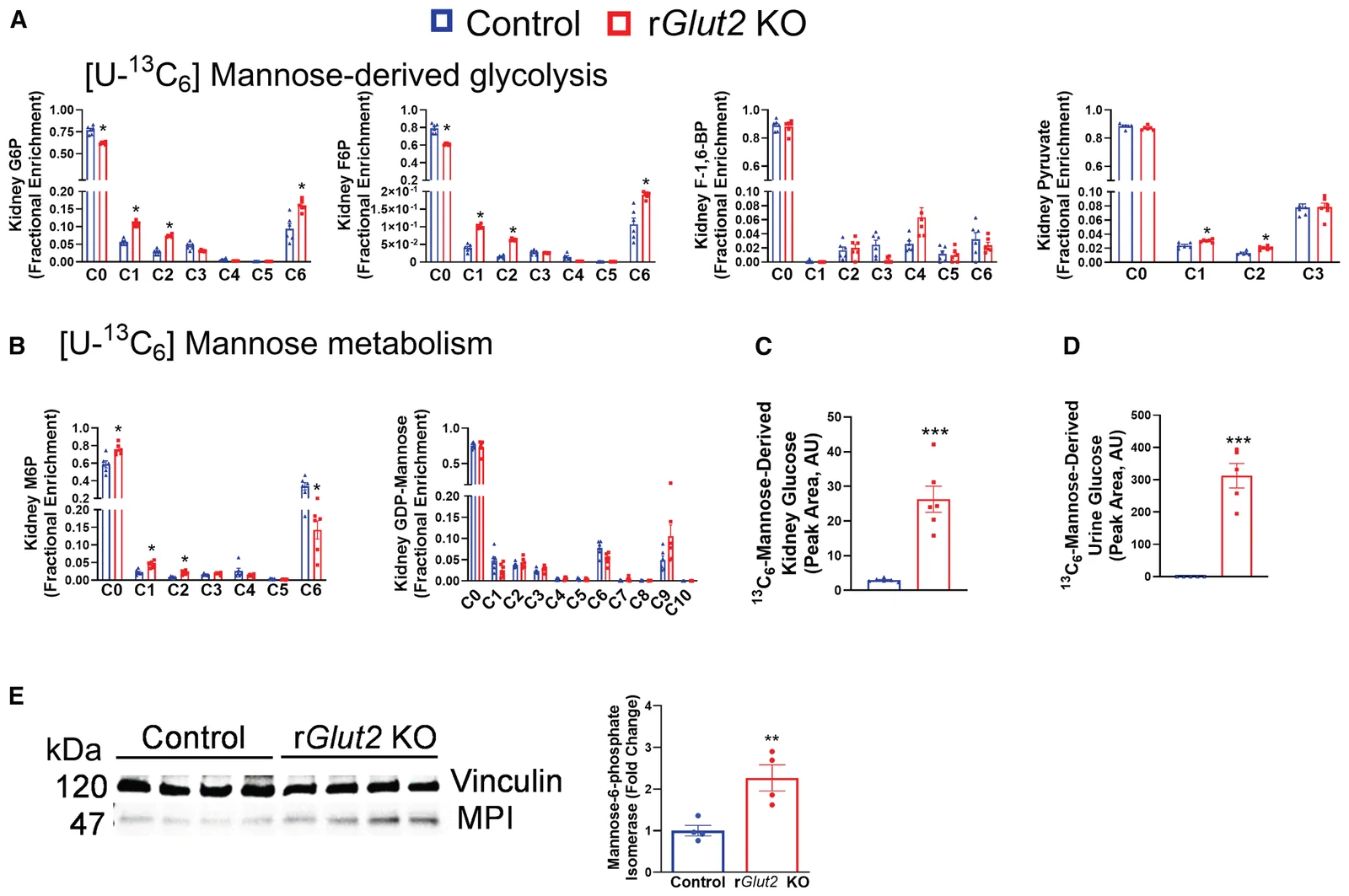
Stable isotope-resolved metabolomics in 14-week-old male r*Glut2* KO mice and their control littermates 60 min after ^13^C_6_-mannose administration, and protein expression analysis of renal mannose-6-phosphate isomerase (MPI) (A) ^13^C-mannose fractional enrichment of kidney hexose-phosphate and glycolytic intermediates G6P, F6P, F-1,6-BP, and pyruvate was increased in r*Glut2* KO kidneys, indicating enhanced mannose entry into the hexose-phosphate pool and glycolytic pathway during glycosuria. (B) ^13^C-mannose fractional enrichment of kidney M6P and GDP-mannose was increased in r*Glut2* KO kidneys. (C) ^13^C-mannose-derived kidney glucose was increased in r*Glut2* KO mice (peak area, AU). (D) ^13^C_6_-mannose-derived urine glucose was increased in r*Glut2* KO mice (peak area, AU), consistent with mannose-derived glucosuria. (E) Representative western blot and quantification showing increased renal MPI protein expression in r*Glut2* KO mice. Vinculin was used as a loading control. Fractional enrichment values represent the fraction of each metabolite derived from ^13^C_6_-mannose, corrected for natural isotope abundance. *N* = 7 (A and B), ***p < 0.001, **p < 0.01, *p < 0.05; multiple comparison unpaired *t* test corrected using Holm-Sidak method (A and B); two-tailed unpaired *t* test (C–E). Data are represented as mean ± SEM.

To directly determine the role of mannose in mediating the glucose-sparing or -producing effects observed during renal glycosuria, we administered ^13^C_6_ mannose (oral gavage, 60 mg/mouse) to r*Glut2* KO mice and their control littermates. We then analyzed glucose as well as mannose metabolism pathways 60 min after the labeled mannose administration. We observed that incorporation of ^13^C-mannose into hexose-phosphate and upper-glycolytic intermediates, including M6P, was higher in the kidneys of the KO mice compared with their littermate controls (Figures 2A and 2B; quantitative metabolite abundances in μmol/g protein are shown in Figures S4C, S4D). The contribution of ^13^C-mannose to kidney GDP-mannose is reduced (Figure 2B) in the KO mice, indicating the accumulation of M6P for gluconeogenic or upstream metabolic pathways.

NMR analysis of ^13^C_6_ mannose tracing showed fractional enrichment of ^13^C-labeled glucose in the urine of r*Glut2* KO mice (~8.5 ± 3%, mean ± SD) but not in the control group (glucose was undetectable in the control mice). Consistent with this, ^13^C_6_-mannose-derived glucose was increased in both the kidney (Figure 2C) and urine (Figure 2D) of r*Glut2* KO mice compared with controls, providing independent confirmation of mannose-to-glucose conversion. Similarly, NMR analysis of plasma from the ^13^C_6_-mannose tracer experiment showed that ^13^C-mannose-derived plasma glucose fractional enrichment was significantly higher in r*Glut2* KO mice compared with controls (13.7 ± 0.8% vs. 10.7 ± 0.9%; *p* = 0.038), providing direct evidence that mannose-to-glucose conversion contributes to the systemic glucose pool *in vivo*. This finding is consistent with conversion of mannose-derived carbon into glucose in KO mice through the pathways delineated in Figure S1. Consistent with upregulation of the enzymatic machinery for glucose-to-mannose conversion, MPI protein expression was significantly increased in r*Glut2* KO kidneys compared with controls (Figure 2E), providing protein-level corroboration of the isotope tracing findings. In addition, ^13^C-mannose-derived glucose (Figure 2C) and lactate (a gluconeogenic substrate) were increased in the kidneys of KO mice compared with control littermates (Figure S3), further supporting a contribution of mannose to renal glucose production during glycosuria. Quantitative metabolite abundances (μmol/g protein) for these mannose-derived intermediates are shown in Figure S4. Together, the ^13^C-mannose tracing and urinary ^13^C-glucose enrichment are consistent with net mannose-to-glucose conversion *in vivo*, supporting substrate-level glucose production through the hexose-phosphate pathway during renal glycosuria. To confirm that mannose-to-glucose conversion is not limited to the fed state, we performed additional ^13^C-mannose tracing following a 6-h fast; kidney and skeletal muscle showed comparable increases in ^13^C-mannose-derived labeling of hexose-phosphate and tricarboxylic acid (TCA) cycle intermediates under fasting conditions (Figures S10 and S11), and ^13^C-mannose-derived plasma glucose was increased in KO mice (Figure S10D), confirming that this pathway is active across nutritional states.

**Figure 3.**
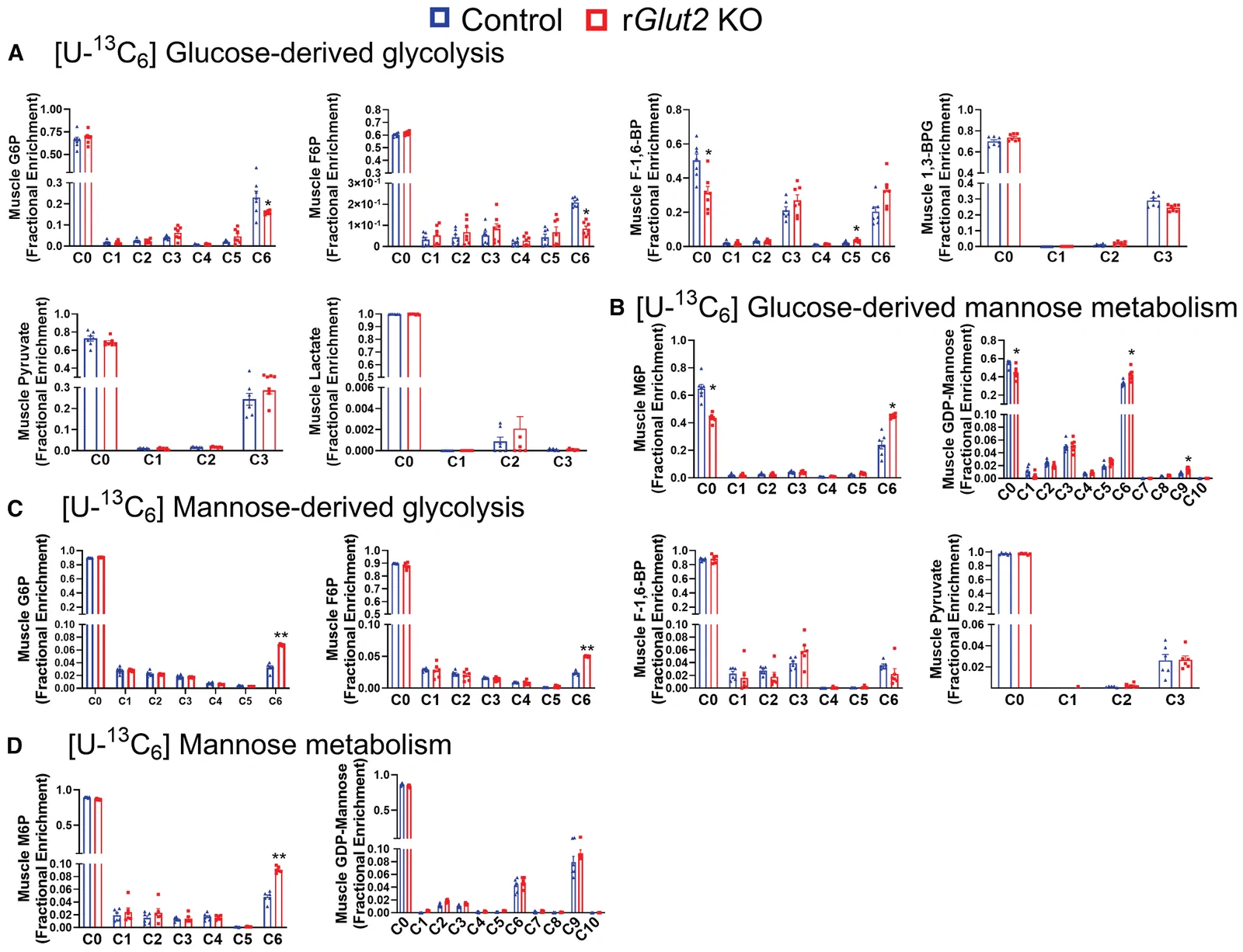
Fractional enrichment of soleus muscle glycolytic and mannose pathway metabolites in 14-week-old male r*Glut2* KO mice and their control littermates 60 min after ^13^C_6_-glucose or ^13^C_6_-mannose administration (A) ^13^C_6_-glucose fractional enrichment of muscle glycolytic intermediates such as G6P and F6P was decreased in r*Glut2* KO mice, indicating suppressed glucose utilization in skeletal muscle during glycosuria. (B) ^13^C_6_-glucose fractional enrichment of muscle mannose pathway metabolites M6P and GDP-mannose was increased in r*Glut2* KO mice. Because M6P is not a direct product of glucose phosphorylation, elevated M+6 M6P in skeletal muscle during the ^13^C_6_-glucose experiment is most consistent with circulating ^13^C_6_-mannose likely derived from kidney MPI-mediated glucose-to-mannose conversion, supporting inter-organ contribution of mannose-derived carbon to skeletal muscle metabolism during glycosuria. (C) ^13^C_6_-mannose fractional enrichment of muscle G6P and F6P was increased in r*Glut2* KO mice, whereas F-1,6-BP and pyruvate were unchanged, indicating increased contribution of mannose-derived carbon to skeletal muscle hexose-phosphate metabolism without progression into lower glycolysis, as glucose utilization is reduced. (D) ^13^C_6_-mannose fractional enrichment of muscle M6P was increased in r*Glut2* KO mice, while GDP-mannose was unchanged.. Fractional enrichment values represent the fraction of each metabolite derived from the indicated tracer, corrected for natural isotope abundance. *N* = 7, **p < 0.01, *p < 0.05, multiple comparison unpaired *t* test corrected using Holm-Sidak method. Data are represented as mean ± SEM.

In the skeletal (soleus) muscle, ^13^C-glucose fractional enrichment of glycolytic intermediates was reduced during glycosuria (Figure 3A; quantitative metabolite abundances in μmol/g protein in Figure S5A), indicating reduced glycolytic flux and/or diminished glucose utilization. Notably, ^13^C-glucose fractional enrichment of mannose pathway metabolites M6P and GDP-mannose was increased in KO skeletal muscle (Figure 3B; quantitative metabolite abundances in μmol/g protein in Figure S5B)—an unexpected finding that implies the labeled M6P originated from circulating ^13^C_6_-mannose released by the kidney via MPI-mediated conversion, rather than from direct muscle glucose utilization, supporting an inter-organ contribution of mannose-derived carbon to skeletal muscle metabolism during glycosuria. By contrast, ^13^C_6_-mannose fractional enrichment of G6P, F6P, and M6P increased (Figures 3C and 3D; quantitative metabolite abundances in μmol/g protein in Figures S5C and S5D), consistent with increased mannose-derived carbon entry into the hexose-phosphate pool. However, the absence of increased F-1,6-BP and pyruvate fractional enrichment (Figure 3C), together with reduced F-1,6-BP abundance (Figure S5C), indicates that mannose carbons do not progress through the phosphofructokinase step into lower glycolysis.. Instead, mannose is largely confined to the upper glycolytic/hexose-phosphate network. These findings are consistent with increased contribution of mannose-derived carbon in skeletal muscle during renal glycosuria.

**Figure 4.**
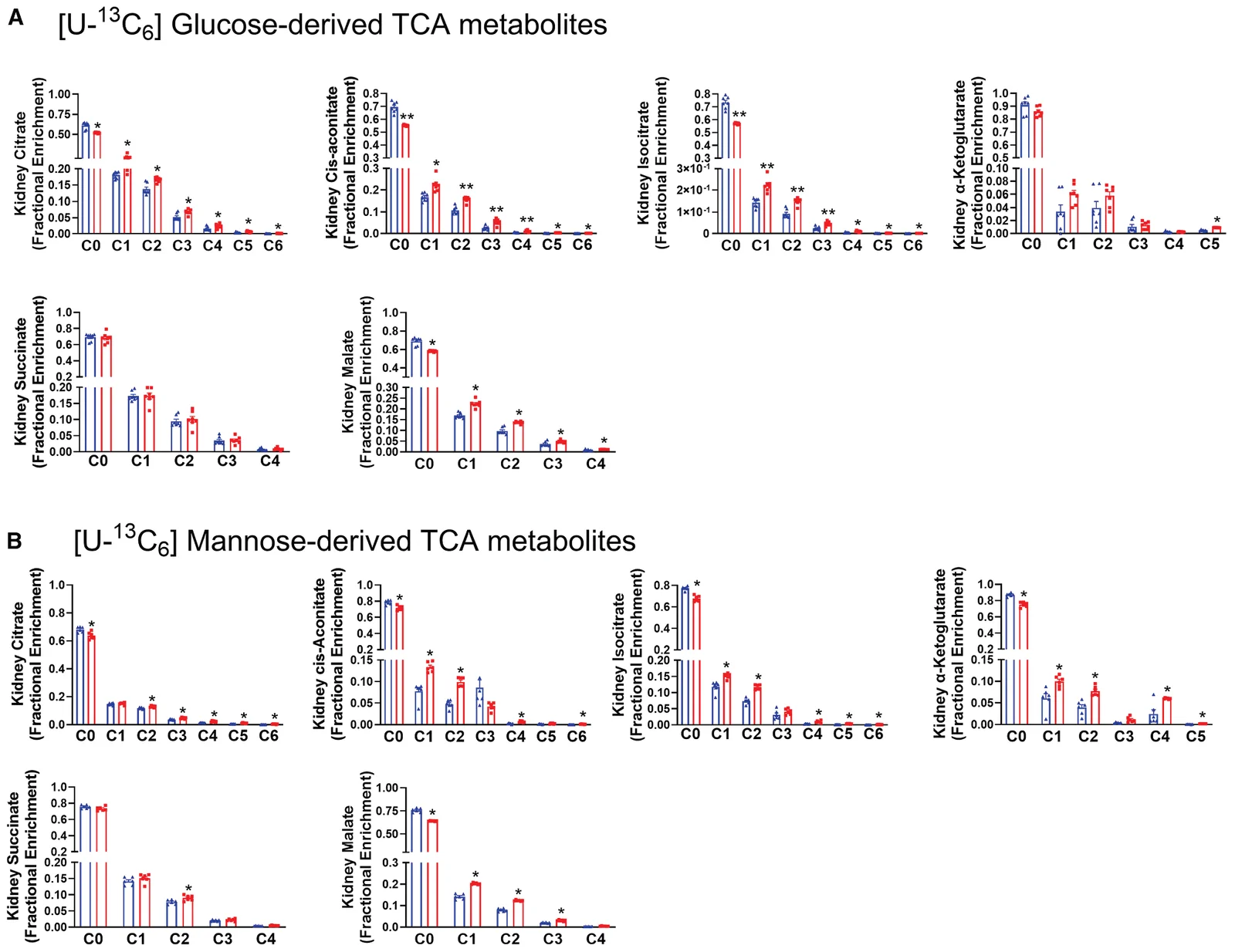
Fractional enrichment of kidney TCA cycle metabolites in 14-week-old male r*Glut2* KO mice and their control littermates 60 min after ^13^C_6_-glucose or ^13^C_6_-mannose administration (A) ^13^C-glucose fractional enrichment of kidney TCA cycle intermediates citrate, *cis*-aconitate, isocitrate, α-ketoglutarate, succinate, and malate was increased in r*Glut2* KO kidneys, consistent with increased contribution of glucose-derived carbon to TCA cycle intermediates during glycosuria. (B) ^13^C-mannose fractional enrichment of kidney TCA cycle intermediates citrate, *cis*-aconitate, isocitrate, α-ketoglutarate, succinate, and malate was also increased in r*Glut2* KO kidneys, consistent with contribution of mannose-derived carbon to renal oxidative metabolism during glycosuria. Fractional enrichment values represent the fraction of each metabolite derived from the indicated tracer, corrected for natural isotope abundance. *N* = 7, **p < 0.01, *p < 0.05, multiple comparison unpaired t test corrected using Holm-Sidak method. Data are represented as mean ± SEM.

**Figure 5.**
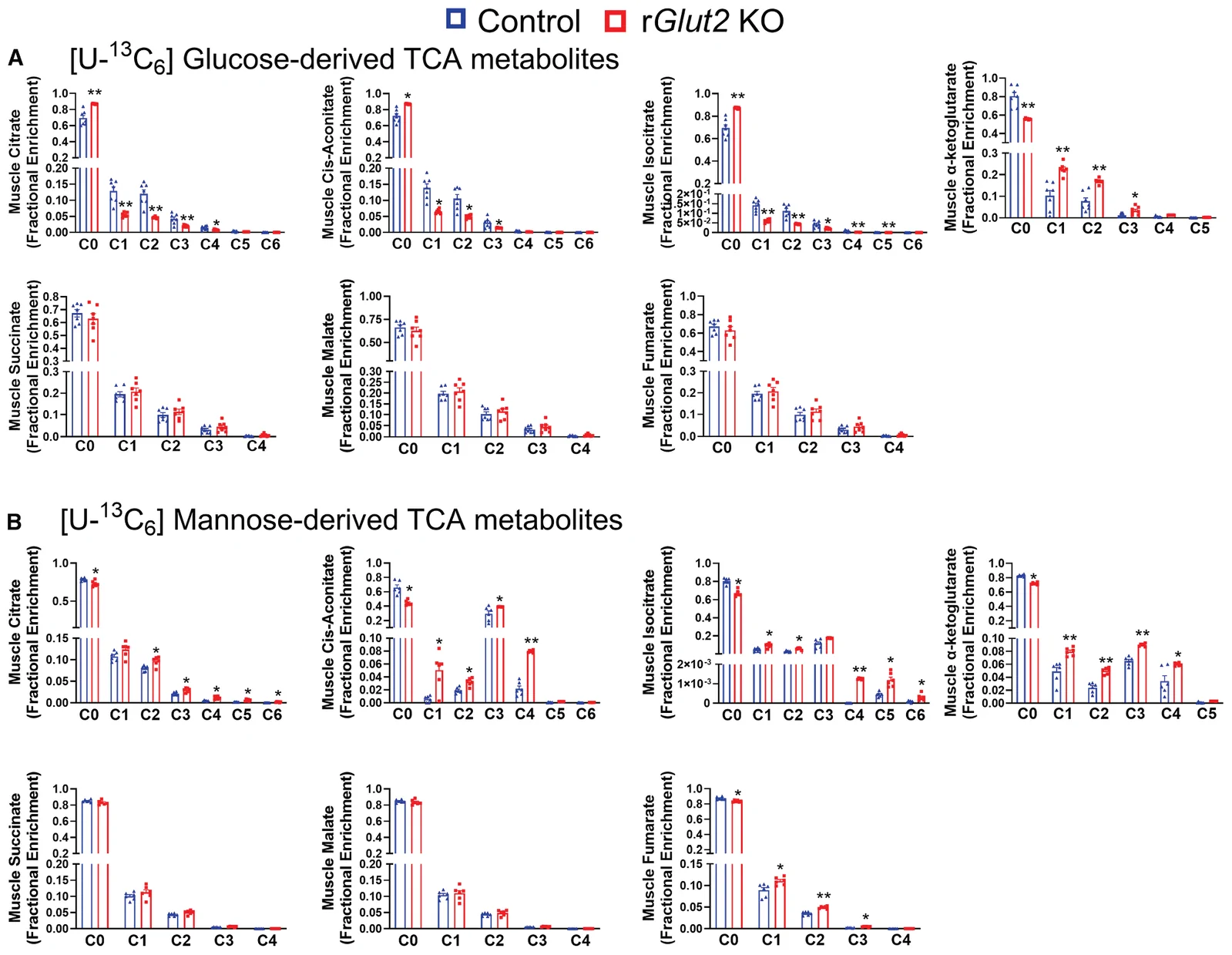
Fractional enrichment of soleus muscle TCA cycle metabolites in 14-week-old male r*Glut2* KO mice and their control littermates 60 min after ^13^C_6_-glucose or ^13^C_6_-mannose administration (A) ^13^C-glucose fractional enrichment of muscle TCA cycle intermediates citrate, *cis*-aconitate, isocitrate, α-ketoglutarate, succinate, malate, and fumarate was reduced or unchanged in r*Glut2* KO soleus muscle, except for α-ketoglutarate which was increased, indicating suppressed glucose oxidation in skeletal muscle during glycosuria. (B) ^13^C-mannose fractional enrichment of muscle TCA cycle intermediates citrate, cis-aconitate, isocitrate, α-ketoglutarate, succinate, malate, and fumarate was increased in rGlut2 KO mice, consistent with increased contribution of mannose-derived carbon to TCA cycle intermediates in skeletal muscle. Fractional enrichment values represent the fraction of each metabolite derived from the indicated tracer, corrected for natural isotope abundance. *N* = 7, **p < 0.01, *p < 0.05, multiple comparison unpaired *t* test corrected using Holm-Sidak method. Data are represented as mean ± SEM.

To determine how these glycolytic shifts affect downstream oxidative metabolism, we examined ^13^C incorporation into TCA-cycle intermediates. In the kidney, fractional enrichment of both ^13^C-glucose and ^13^C-mannose into TCA-cycle metabolites increased during renal glycosuria (Figure 4; quantitative metabolite abundances in μmol/g protein in Figure S6). The expected propagation of ^13^C-glucose-derived carbon through sequential TCA cycle turns, including the origin of the distributed citrate isotopologue pattern (M+2, M+4, M+5, and M+6), is illustrated in Figure S2. Conversely, in skeletal muscle, ^13^C fractional enrichment into TCA-cycle intermediates increased only after ^13^C-mannose administration (Figure 5; quantitative data in Figure S7). The skeletal muscle α-ketoglutarate was increased under both tracer conditions (Figure 5), likely reflecting anaplerotic inputs from amino acids such as glutamine and glutamate. In the kidney, increased fractional labeling of upstream TCA intermediates and elevated α-ketoglutarate are similarly consistent with enhanced glutamine-supported anaplerosis during renal glycosuria. These TCA-cycle findings align with the glycolysis results described above, consistent with increased contribution of mannose-derived carbon to oxidative metabolism in skeletal muscle. Likewise, the combined findings from Figures 1E and 4B suggest that renal glucose is routed through the mannose/M6P pathway to support TCA-cycle activity during glycosuria.

Unexpectedly, the liver did not show major changes in the glucose and mannose metabolism at the fed-state timepoint used in this study (Figures S8; S9). To determine whether the liver contributes to mannose-mediated glucose compensation under conditions that favor gluconeogenesis, we performed additional ^13^C-mannose tracing following a 6-h fast. Under fasting conditions, ^13^C-mannose-derived labeling of hepatic glycolytic and TCA cycle intermediates was similarly modest (Figure S12), suggesting that the liver is not the primary site of mannose-to-glucose conversion during renal glycosuria at the timepoint used in this study. The liver plays a key role in gluconeogenesis during fasting, including in r*Glut2* KO mice, as reported previously.^12^ However, because ^13^C_6_ glucose was administered in this study, the liver may have perceived a fed state at the time of metabolic analysis, potentially attenuating detectable changes in glucose metabolism. By contrast, the kidney exhibited marked metabolic alterations in response to glycosuria.

Collectively, the IC-UHR-FTMS and NMR analyses indicate that mannose contributes to maintenance of glucose homeostasis during renal glycosuria by supplying carbon to the hexose-phosphate pool for substrate-level glucose production in the kidney and by supporting oxidative metabolism in skeletal muscle.

### Renal glycosuria induces systemic glucose-sparing physiology

**Figure 6.**
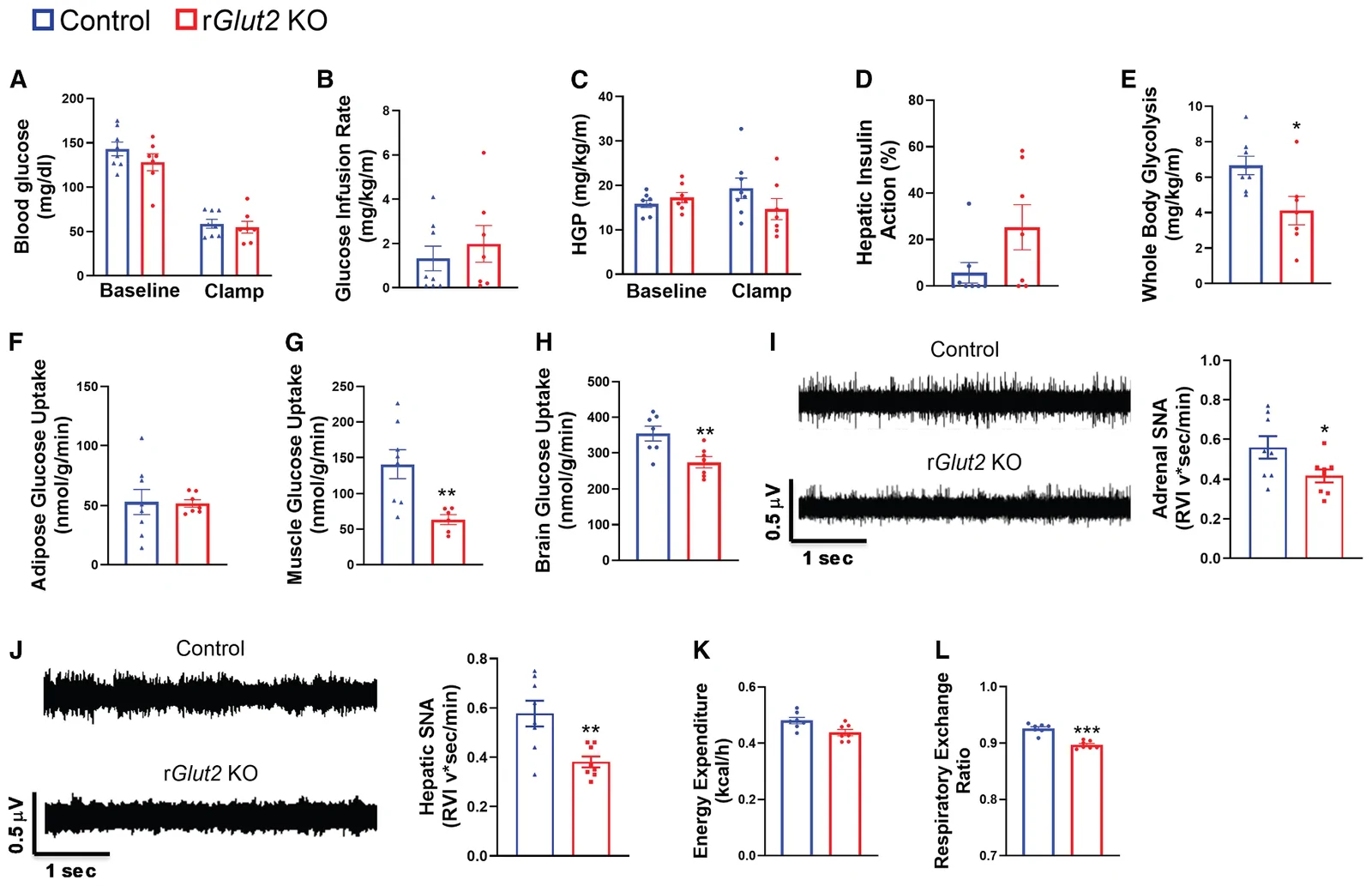
Hyperinsulinemic-hypoglycemic clamp and sympathetic nerve activity (SNA) measurements in 12-week-old male renal glucose transporter 2 knockout (r*Glut2* KO) mice and their control littermates (A) Blood glucose levels during baseline and hypoglycemic clamp. (B) GIR, which was unchanged between groups. (C and D) Hepatic glucose production (HGP) and hepatic insulin action, showing preserved hepatic insulin sensitivity in r*Glut2* KO mice. (E) Whole-body glycolysis was reduced in r*Glut2* KO mice. (F–H) Tissue-specific glucose uptake: unchanged in epididymal white adipose tissue (F), reduced in gastrocnemius muscle (G), and reduced in the brain (H), consistent with tissue-specific insulin resistance. (I and J) Adrenal (I) and hepatic (J) SNA were reduced in r*Glut2* KO mice; representative SNA tracings are shown on the left and quantification on the right. *N* = 7 or 8, *p < 0.05, **p < 0.01, two-tailed unpaired *t* test. (K) Total energy expenditure (kcal/h) was not significantly different between groups (*p* = 0.069, mass-adjusted GLM). (L) Respiratory exchange ratio (RER; VCO_2_/VO_2_; dimensionless) was significantly reduced in r*Glut2* KO mice compared with controls (***p < 0.001, GLM group effect), indicating reduced net carbohydrate oxidation consistent with diversion of glucose and mannose carbons toward gluconeogenesis rather than complete oxidation. RER is inherently mass-independent and requires no body mass covariate. EE statistics from mass-adjusted GLM; RER statistics from GLM group effect only. Data are represented as mean ± SEM.

To determine whether renal glycosuria triggers systemic metabolic adaptations that facilitate glucose sparing, we examined whole-body glucose metabolism and autonomic activity. Hyperinsulinemic-hypoglycemic clamps (Figure 6A) showed that r*Glut2* KO mice maintained the target clamp glucose with the same exogenous glucose infusion rate as controls (GIR unchanged, Figure 6B), indicating that compensatory metabolic adaptations offset urinary glucose loss. The KO mice show normal hepatic insulin sensitivity (Figures 6C and 6D); however, the whole-body glycolysis is reduced (Figure 6E). While the glucose uptake is unaffected in the epididymal white adipose tissue (Figure 6F), we observed a decrease in glucose utilization (insulin resistance) in the gastrocnemius muscle (Figure 6G) and brain (Figure 6H) of the KO mice. The tissue-specific insulin resistance may explain why glucose fractional enrichment was reduced (Figure 3A) and mannose fractional enrichment was higher (Figures 3C and 3D; Figure 5B) in the skeletal muscle of r*Glut2* KO mice compared with the control group. These systemic glucose-sparing adaptations in r*Glut2* KO mice may clarify how glucose is made available for necessary biological activities including the counter-regulatory response.

Because sympathetic nerve activity contributes to counter-regulation during hypoglycemia, we also measured hepatic and adrenal sympathetic output. Both signals were markedly suppressed in r*Glut2* KO mice (Figures 6I and 6J), consistent with a systemic energy-conserving phenotype. These findings support the view that renal glycosuria elicits physiological responses that reduce whole-body glucose utilization and complement the substrate-level rewiring observed in this study. Consistent with substrate switching rather than a global reduction in energy demand, total energy expenditure was not significantly different between r*Glut2* KO and control mice (*p* = 0.069, mass-adjusted GLM; Figure 6K). Notably, the respiratory exchange ratio (RER) was significantly reduced in r*Glut2* KO mice (*p* < 0.001; Figure 6L), reflecting reduced net carbohydrate oxidation to CO_2_. This is consistent with diversion of glucose and mannose carbons toward gluconeogenic substrate pools rather than complete oxidative metabolism—and is not contradictory to the increased mannose utilization described above, since mannose in r*Glut2* KO mice is routed toward gluconeogenesis rather than fully oxidized. Body mass and food intake did not differ significantly between groups, as previously reported,^2^ confirming that the phenotype reflects substrate switching rather than differences in energy intake or body composition. Together, these findings demonstrate that r*Glut2* KO mice maintain glucose homeostasis during chronic renal glycosuria through a coordinated shift in substrate utilization, whereby reduced glucose oxidation is compensated by increased mannose and glutamine metabolism, without any change in total energy expenditure.

### Mannose and glutamine contribute to glucose compensation during renal glycosuria

Given that renal glycosuria increased mannose and glutamine levels as described above, we next determined whether inhibiting mannose production or blocking glutamine metabolism would impair glucose homeostasis during renal glycosuria.

**Figure 7.**
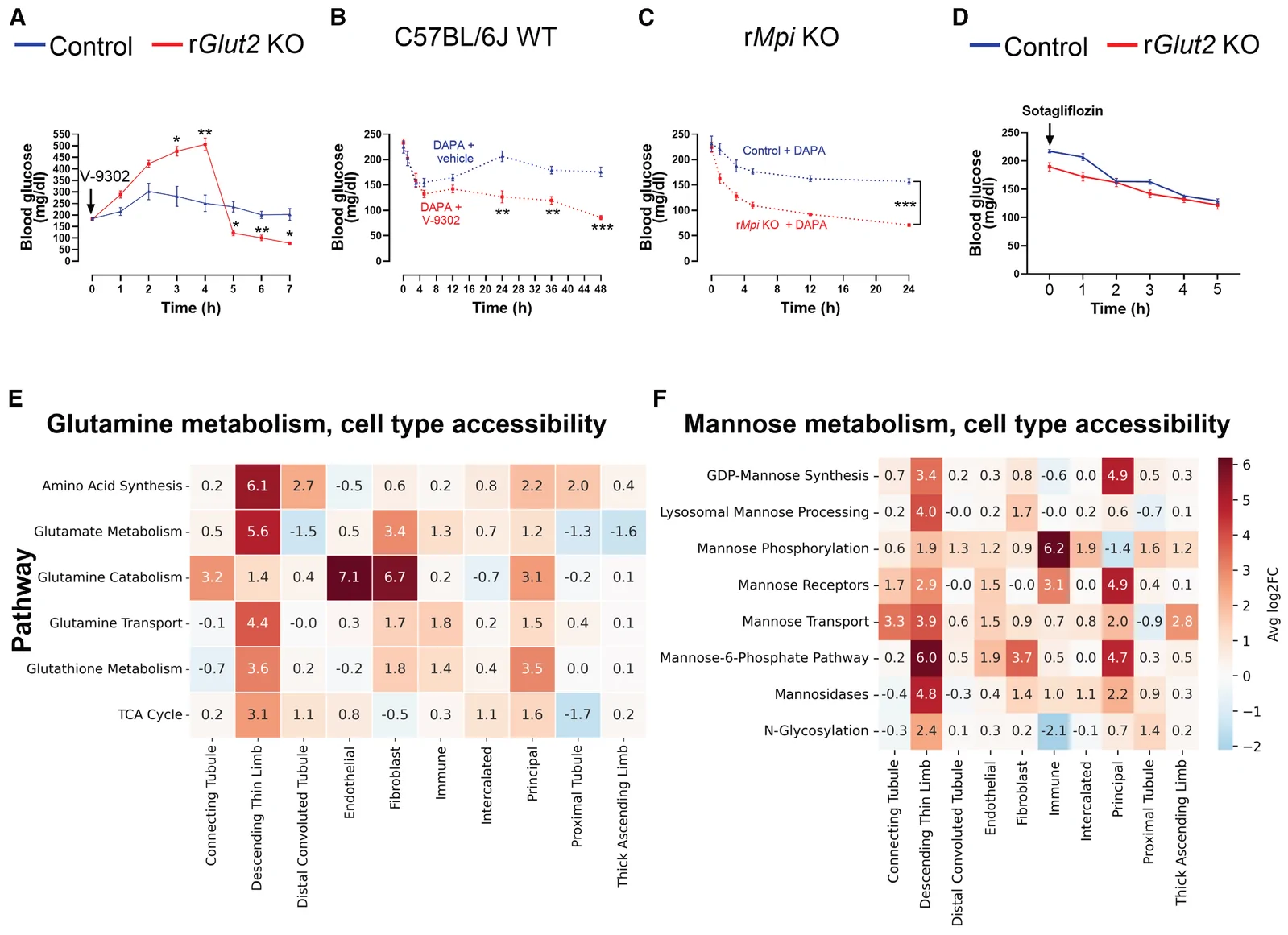
Effects of V-9302 (an inhibitor of glutamine transport/metabolism), dapagliflozin (DAPA; SGLT2 inhibitor), and sotagliflozin (dual SGLT1/SGLT2 inhibitor) on blood glucose levels, and chromatin accessibility of mannose and glutamine metabolic pathways (A) In 26-week-old male r*Glut2* KO mice, V-9302 (75 mg/kg) induced hypoglycemia following an initial rise in blood glucose. (B) In 9-week-old WT mice treated with DAPA (10 mg/kg, twice daily), V-9302 (75 mg/kg, once daily; 1 h after the second DAPA dose) produced a similar hypoglycemic effect. (C) DAPA induced hypoglycemia in 20-week-old AAV-mediated renal mannose-6-phosphate isomerase knockout (r*Mpi* KO) mice. (D) Sotagliflozin (30 mg/kg) reduced blood glucose similarly in control and r*Glut2* KO mice. (E and F) ATAC-seq analysis of kidneys from 12-week-old male r*Glut2* KO and control mice showed increased chromatin accessibility in glutamine (E) and mannose (F) metabolic pathways, consistent with enhanced substrate availability. N = 6 (A–D); n = 3 per group (E and F). *p < 0.05, **p < 0.01, ***p < 0.001; repeated-measures two-way ANOVA with Bonferroni post hoc correction. Differential accessibility in (E and F) was assessed by Wilcoxon rank-sum test with FDR-adjusted p < 0.05.

We administered V-9302^23^—a glutamine transporter ASCT2 (encoded by *Slc1a5* gene) inhibitor—to the mice to determine whether glutamine metabolism contributes to glucose compensation in renal glycosuria. We monitored blood glucose levels at different times following the V-9302 administration (Figure 7A). Remarkably, after the initial increase in blood glucose levels, the glucose concentration in the r*Glut2* KO mice decreased to potentially life-threatening hypoglycemia compared with the control mice. The KO mice had to be given a glucose bolus (60 mg/mouse, intraperitoneally) to restore normal conditions. The initial increase in the blood glucose levels was likely due to a concomitant increase in circulating glutamine levels, which eventually decrease with time, as reported previously.^23^ Next, we administered V-9302 (once a day) or its vehicle to C57BL6/J wildtype mice (000664, the Jackson laboratory) that were previously treated with the SGLT2 inhibitor dapagliflozin (twice a day), and monitored their blood glucose levels. V-9302 was administered one hour after the second dose of dapagliflozin for two days. The V-9302 and dapagliflozin treated mice showed hypoglycemia compared with the mice that received the vehicle and dapagliflozin (Figure 7B), thereby supporting the findings from V-9302-treated r*Glut2* KO mice. These functional findings are corroborated by direct ^13^C-glutamine tracing, which showed increased glutamine-derived carbon incorporation into TCA cycle intermediates and gluconeogenic precursors in r*Glut2* KO kidneys compared with controls (Figure S13), consistent with enhanced glutamine-supported anaplerosis driving gluconeogenic substrate supply. Although V-9302 has activity at additional amino acid transporters beyond ASCT2, the convergent evidence from pharmacological inhibition and direct isotope tracing supports a specific role for glutamine in glucose compensation during renal glycosuria.

To block renal mannose production, we deleted *Mpi* in the kidney of *Mpi*^flox/flox^ mice by delivering AAV9-CMV-Cre via retrograde renal pelvis injection; AAV9-CMV-GFP was used as the control. Three weeks after AAV administration, mice were treated with dapagliflozin (twice a day) and blood glucose levels were monitored as described above. *Mpi* deficient mice exhibited hypoglycemia compared with their control group (Figure 7C). Renal *Mpi* deletion was confirmed by RT-qPCR (6.3 ± 2% of control expression; controls: 100 ± 31%; *n* = 7, mean ± SEM) at the end of the study. These findings indicate that mannose contributes to maintaining glucose homeostasis during renal glycosuria.

Because kidney SGLT1 is assumed to compensate for SGLT2 inhibition,^5^ a dual SGLT1 and SGLT2 inhibitor could improve the efficiency of SGLT2 inhibition alone. To test this hypothesis, we monitored blood glucose levels in r*Glut2* KO mice after administering sotagliflozin (dual SGLT1 and SGLT2 inhibitor). The drug reduced blood glucose levels in both the KO mice and their control littermates to a similar extent (Figure 7D). This preliminary observation is consistent with the possibility that glucose reabsorption by kidney SGLT1 does not fully account for how r*Glut2* KO mice maintain normal blood glucose levels in renal glycosuria, although single-dose pharmacokinetics and potential off-target effects of sotagliflozin preclude a definitive conclusion. We have previously shown that kidney SGLT2 is reduced, but SGLT1 is unaffected, in r*Glut2* KO mice.^2^ Altogether, these data indicate that mannose utilization and glutamine metabolism play an important role in supporting glucose compensation during renal glycosuria. To directly assess the contribution of glutamine-derived carbon to renal and systemic metabolism during glycosuria, we performed ^13^C-glutamine tracing following a 6-h fast. In KO kidneys, ^13^C-glutamine incorporation into TCA cycle intermediates and gluconeogenic precursors was increased relative to controls (Figure S13), consistent with enhanced glutamine-supported anaplerosis and gluconeogenic substrate supply. Comparable changes were observed in skeletal muscle (Figure S14), while hepatic ^13^C-glutamine labeling did not differ significantly between groups (Figure S15), further delineating the kidney as the primary site of glutamine-mediated metabolic adaptation during renal glycosuria.

### Kidney molecular adaptations and systemic stress during renal glycosuria

To determine whether molecular changes in the kidney contribute to the metabolic adaptations observed *in vivo*, we analyzed kidney tissue using single-nucleus RNA-seq, ATAC-seq, and reduced-representation bisulfite sequencing (RRBS). RNA-seq revealed reduced expression of the glutamine-catabolism gene *Gls*, along with the glutamate-metabolism genes *Got2* and *Glud1*, in r*Glut2* KO kidneys (Figures 7E and Data S2), and RRBS showed hypermethylation of *Got2* (Data S4). In parallel, the glutamine transporter *Slc1a5* was upregulated, with higher chromatin accessibility (ATAC-seq) and promoter hypomethylation, consistent with enhanced glutamine uptake (Figures 7E and Data S3 and S4). Notably, glutaminase-locus accessibility was increased despite reduced *Gls* transcription (Figure 7E), consistent with substrate- and mass-action-driven catabolic flux rather than transcriptional control. Similarly, genes involved in mannose metabolism, including *Mpi*, exhibited increased accessibility and the mannose transporter gene *Slc5a10* was upregulated in the KO mice (Figures 7F and Data S2 and S3). *Mpi* encodes mannose 6-phosphate isomerase, which interconverts mannose-6-phosphate and fructose-6-phosphate, supporting the glucose and mannose shunt identified in the ^13^C-tracing studies. Because transcript abundance need not reflect enzyme activity or metabolic flux—which is governed largely by substrate availability and mass action—these transcriptional and epigenetic changes are best interpreted alongside the ^13^C-glutamine tracing data (Figures S13-S15). The tracing shows increased entry of glutamine-derived carbon into TCA-cycle and gluconeogenic intermediates in r*Glut2* KO kidneys, consistent with enhanced glutamine uptake supplying anaplerotic carbon—via glutaminase-mediated hydrolysis of glutamine to glutamate, transamination to α-ketoglutarate, and oxidative TCA-cycle metabolism to oxaloacetate—for gluconeogenic precursor supply rather than complete oxidation to CO_2_ for energy production.

To further assess systemic signals associated with glucose sparing, we measured circulating cytokines using a microarray dot plot. Several markers of metabolic stress and immune activation, including CCL6, GDF15, endostatin, and IGFBP1, were elevated in r*Glut2* KO mice, whereas cytokines associated with immunosuppressive signaling such as VEGF, RAGE, and PDGF-BB were reduced (Figure S16). These results extend our previous findings that renal glycosuria elicits an acute phase response including activation of the immune system.^12^ Collectively, these changes are consistent with systemic metabolic stress and immune alterations, which may contribute to the kidney’s adaptive metabolic rewiring in response to glucose loss.

To evaluate whether classical gluconeogenic genes were altered, we examined *Pck1* and *G6pc* in our transcriptomic and ATAC-seq datasets (Data S2 and S3). In the RNA-seq analysis, *Pck*1 was not detectable in any kidney cell types and *G6pc* was reduced in proximal tubule cells of r*Glut2* KO mice (log2FC – 1.90; p adj. = 3.4 × 10^−24^). ATAC-seq analysis aligned with these findings: neither locus showed increased chromatin accessibility under the experimental condition. Instead, both *Pck1* and *G6pc* peaks showed slightly higher accessibility in the control group (fold changes 0.60 and 0.67). These data indicate that changes in *Pck1* or *G6pc* transcription or accessibility are unlikely to explain the substrate-level glucose production observed in our ^13^C-tracing experiments. Therefore, our findings support the conclusions reported previously that the kidney adjusts its gluconeogenic activity largely through changes in substrate delivery, rather than through major transcriptional upregulation of *Pck1* or *G6pc*.^20,24^ Notably, metabolic flux studies demonstrate that *Pck1* (which encodes PEPCK-C) expression does not necessarily predict gluconeogenic flux.^25^

Overall, the molecular analyses suggest that renal glycosuria triggers a coordinated metabolic reprogramming - favoring mannose and glutamine accumulation, reduced transcription of catabolic enzymes, and increased substrate availability to support glucose homeostasis. These findings align with the *in vivo* metabolic flux studies and indicate that substrate-level regulation is a key mechanism of glucose compensation during renal glycosuria.

## DISCUSSION

The glucose lowering efficacy of SGLT2 inhibitors is attenuated by compensatory mechanisms that maintain circulating glucose levels, including endogenous glucose production and possible contributions from residual SGLT1-mediated reabsorption.^5,13^ Prior work indicates that SGLT2 inhibition activates metabolic programs resembling starvation, promoting alternative energy use and conservation in models of type 2 diabetes.^26,27^ Similarly, mice constitutively lacking *Glut2* in kidney proximal tubule cells show an energy-conserving phenotype in response to renal glycosuria.^28^ However, the metabolic pathways that sustain glucose homeostasis during chronic renal glucose loss remain unclear.

Here, we investigated the metabolic pathways and associated molecular mechanisms that facilitate glucose compensation in renal glycosuria. To reduce potential confounding developmental effects associated with constitutive KO models, we used a tamoxifen-inducible r*Glut2* KO mouse model.^2^ Following renal *Glut2* deletion, these mice develop marked glycosuria accompanied by secondary loss of *Slc5a2* expression, resulting in sustained renal glucose wasting. This model therefore provides a genetic system to examine metabolic adaptations that support systemic glucose homeostasis during chronic renal glycosuria in adult mice. Metabolic profiling and stable isotope tracing revealed coordinated adaptations in mannose and glutamine metabolism that support glucose compensation.

Mannose metabolism has been described as an auxiliary energy pathway activated under glucose scarcity or high energy demand^17-19^ Our present study shows that mannose metabolism is upregulated during renal glycosuria, and contributes carbon to upper-glycolytic intermediates and to substrate-level glucose production via the hexose-phosphate pathway, thereby helping sustain glucose homeostasis. Elevated mannose may also contribute to tissue-specific insulin resistance, consistent with prior associations^29^ and with the reduced muscle glucose utilization observed in r*Glut2* KO mice. Together, these adaptations support a role for mannose as a substrate contributing to renal and systemic glucose sparing during glycosuria.

Glutamine is a major anaplerotic substrate that supports energy metabolism and precursor supply for glucose formation under conditions of metabolic stress.^20-22^ In r*Glut2* KO mice, renal glutamine levels were elevated, and inhibition of glutamine transport with V-9302 caused hypoglycemia in both r*Glut2* KO and dapagliflozin-treated mice. These findings indicate that glutamine availability contributes to maintaining glucose homeostasis during glycosuria. In addition, the increase in renal α-ketoglutarate during glycosuria is consistent with enhanced amino-acid-supported anaplerosis, as glutamine is well-established to be converted to glutamate and then to α-ketoglutarate for entry into the TCA cycle.^21,30^ Together, these data identify glutamine metabolism as a critical component of the substrate-level adaptations that sustain glucose compensation during renal glucose loss.

Hormonal mediators of compensatory glucose production during glycosuria remain debated. Although early studies implicated glucagon secretion,^31^ subsequent work^32^—including ours^2^—found no clear evidence for elevated glucagon following renal *Glut2* deletion or SGLT2 inhibition. Instead, glycosuric mice exhibit increased corticosterone and markers of metabolic stress.^12^ In the present study, our data indicate that substrate-level metabolic adaptations involving mannose and glutamine play an important role in maintaining glucose homeostasis during glycosuria.

Importantly, SGLT2 inhibitors lower glucose without causing hypoglycemia in type 2 diabetes—likely because the metabolic pathways identified here maintain glucose homeostasis despite urinary glucose loss. These pathways provide a mechanistic basis for the preserved euglycemia observed during glycosuria and may help inform strategies to optimize the glucose-lowering efficacy of SGLT2 inhibitors while maintaining metabolic safety. Future work should define the kidney-derived signals that communicate renal glycosuria to the liver, muscle, and brain to coordinate systemic glucose sparing.

### Limitations of the study

All experiments were conducted in non-diabetic mice with normal body weight and glucose levels to define physiological compensation to renal glycosuria. Whether mannose and glutamine metabolism make similar contributions in the context of obesity or diabetes remains to be determined. Although mannose is predominantly transported by SGLT4^33^ and SGLT5,^34^ it is possible that lack of renal GLUT2 and SGLT2 may have indirectly altered mannose transport and metabolism in r*Glut2* KO mice. Similarly, SGLT4 and SGLT5 may contribute to glucose reabsorption in renal glycosuria as these transporters exhibit low affinity for glucose. Use of specific mannose transport inhibitors or kidney-specific *Mpi* KO models will further help clarify these possibilities. Additional studies are also needed to identify the renal cell types responsible for mannose production and to define the signals that induce mannose and glutamine metabolism during renal glycosuria. V-9302 inhibits additional amino acid transporters beyond ASCT2,^23^ so its effects may extend beyond glutamine transport; however, this limitation is partially addressed by the direct ^13^C-glutamine tracing data (Figures S13-S15), which demonstrate glutamine-derived carbon entry into gluconeogenic intermediates specifically in r*Glut2* KO kidneys. Glucose-stimulated insulin secretion has previously been shown to be comparable between r*Glut2* KO and control mice,^2^ indicating that the metabolic adaptations described here are unlikely to be secondary to differences in insulin secretion. Finally, we did not directly quantify contributions from individual gluconeogenic amino acids, lactate, or glycogenolysis to endogenous glucose production.

## STAR★METHODS

### EXPERIMENTAL MODEL AND STUDY PARTICIPANT DETAILS

#### Mouse models

The r*Glut2* KO mouse model (tamoxifen-inducible, kidney-specific *Glut2* deletion) has been described previously.^2,12^ Following tamoxifen induction, r*Glut2* KO mice develop progressive loss of *Slc5a2* (SGLT2) expression and marked urinary glucose excretion while maintaining normoglycemia.

r*Glut2* KO mice and control littermates were used in all experiments. Ages and sexes are specified in figure legends. All experiments used male mice; male and female r*Glut2* KO mice exhibit comparable metabolic phenotypes.^2,12^, justifying the single-sex design. This is acknowledged as a limitation.

For the renal *Mpi* knockout model, adult *Mpi*^flox/flox^ mice (T016292, GemPharmatech) were used. C57BL/6J wildtype mice (Cat#000664, The Jackson Laboratory; RRID: IMSR_JAX:000664) were used for pharmacological experiments.

After genotyping, mice were randomly assigned to experimental groups using GraphPad Prism 10.2.3. Researchers were not blinded to group allocation owing to obvious phenotypic differences (polyuria, polydipsia); this is acknowledged as a limitation. No animals or data points were excluded.

All animal studies were approved by the Institutional Animal Care and Use Committees of the University of Kentucky, University of Iowa, and University of Massachusetts, and conducted in accordance with NIH guidelines.

### METHOD DETAILS

#### Untargeted metabolomics and stable isotope-resolved metabolomics (SIRM) analysis

r*Glut2* KO mice and their control littermates^2,12^ were used in this study. We have described the production, genotyping of r*Glut2* KO and their control mice previously.^2^ Randomization and blinding procedures are described in the Experimental Model section. The age and sex of mice included in each experiment in this study are mentioned in the figure legends. No mice or data points were excluded from the analysis. We did not repeat all the experiments in both sexes because our previous study^2,12^ demonstrated that male and female r*Glut2* KO mice exhibited a similar phenotype compared to their corresponding control groups in the context of glucose homeostasis. All mouse studies were approved by University of Kentucky, University of Iowa, or University of Massachusetts.

Primary metabolism in mouse kidneys was analyzed using GC-TOF-MS (West coast metabolomics center, UC Davis). The kidney samples were collected around 9a.m. from non-fasted male mice and flash frozen in liquid nitrogen. The samples were then processed for metabolism analysis as described previously.^37^ For SIRM analysis, ^13^C_6_ glucose (CLM-1396-0.25, Cambridge Isotope Laboratories, USA) or ^13^C_6_ mannose (CLM-6567-0.25, Cambridge Isotope Laboratories, USA) was administered by oral gavage (60 mg/mouse, the isotopes were dissolved in water with the concentration of 200 mg/mL and 300 μL was administered to each mouse) to separate cohorts of mice. Equal dosing was used to ensure comparable tracer exposure across animals. The rationale for administering equal amounts of glucose or mannose to each mouse has been described previously.^9^ The mice were euthanized by decapitation 60 min after the stable isotope administration to collect trunk blood and tissues in this order: urine, trunk blood, kidney, liver, and skeletal muscle (all samples were collected within 6 min of euthanizing the mice). The samples were flash frozen in liquid nitrogen, stored at −80°C until analyzed as described previously.^38^ Ion chromatography coupled with ultra-high resolution Fourier transform mass spectrometry (IC-UHR-FTMS) was used to quantify glucose and mannose metabolites. The frozen tissue samples were pulverized to a fine powder under liquid nitrogen prior to 3-phase extraction. Polar/non-polar metabolites and proteins were extracted simultaneously using acetonitrile:H2O:chloroform (2:1.5:1, v/v) solvent partitioning method as described previously.^39^

IC-UHR-FTMS was carried out as described previously.^40^ Metabolites were separated on a Thermo Scientific Dionex IonPac AG11-HC analytical column paired with a Dionex IonPac AS11-HC guard column in a Dionex ICS-5000+ DP Ion Chromatography system interfaced to a Thermo Fisher Orbitrap Fusion Tribrid mass spectrometer (Waltham, MA, USA) run in negative MS1 mode at a resolving power of 500,000 at m/z 200 and an m/z range of 80–800. Data were analyzed using TraceFinder software and an in-house database. MS1 peak areas of assigned metabolites and isotopologues were corrected for natural abundance as reported previously^35,40^ and quantified against a calibration standard mixture. Metabolite amounts thus obtained were normalized to the amount of extracted protein and is reported as μmol/g protein. Protein was quantified using the bicinchoninic acid assay.

To assess tracer metabolism under gluconeogenic conditions, separate cohorts of r*Glut2* KO mice and control littermates were fasted for 6 h (8 a.m.–2 p.m.) prior to tracer administration. For fasted ^13^C-mannose experiments, ^13^C_6_-mannose (60 mg/mouse, dissolved in water as described above, oral gavage) was administered and tissues collected 60 min later as described above. For ^13^C-glutamine experiments, ^13^C_5_-glutamine (30 mg/mouse, dissolved in sterile 0.9% normal saline, intraperitoneally, CLM-1822-H-0.25, Cambridge Isotope Laboratories) was administered and tissues were collected 60 min after injection. Kidney, liver, soleus muscle, and plasma were analyzed by IC-UHR-FTMS as described above.

#### Natural isotope abundance correction and fractional enrichment calculation

MS1 peak areas of assigned metabolites and isotopologues were corrected for natural isotope abundance (NIA) using the TraceFinder pipeline as described previously,^35,40^ and applied uniformly to all metabolites and isotopologues across all groups prior to any comparative analysis. This correction is described in detail in Sun et al.^40^ and the IsoCor framework.^35^

Fractional mass isotopomer distributions (MIDs) represent the proportion of each metabolite pool derived from the ^13^C-labeled tracer following natural isotope abundance correction. Quantitative metabolite pool sizes (μmol/g protein) are presented separately in the corresponding Figures S4-S9 to distinguish changes in metabolic flux from changes in total metabolite abundance. Presenting these two metrics separately directly addresses whether observed differences reflect isotopic labeling patterns or changes in pool size.

#### Plasma ^13^C enrichment measurement

Plasma ^13^C tracer enrichment was measured from trunk blood collected at euthanasia across all SIRM experiments to confirm equal tracer delivery between r*Glut2* KO and control groups. For the fed-state ^13^C_6_-glucose tracer experiments, plasma ^13^C-glucose fractional enrichment was measured by NMR (Presat) and was 32.2 ± 2.4% (Control) vs. 40.1 ± 3.2% (r*Glut2* KO; *p* = 0.074, ns). For the fed-state ^13^C_6_-mannose tracer experiments, plasma mannose fractional enrichment was 97.1 ± 0.2% (Control) vs. 97.7 ± 0.3% (r*Glut2* KO; *p* = 0.089, ns), confirming equivalent mannose tracer delivery. Notably, plasma glucose derived from the ^13^C_6_-mannose tracer was significantly higher in r*Glut2* KO mice (13.7 ± 0.8% vs. 10.7 ± 0.9%; *p* = 0.038), consistent with enhanced mannose-to-glucose conversion *in vivo*. For the fasted-state ^13^C_6_-mannose tracer experiments, plasma M+6 enrichment was 86.8 ± 2.1% (Control) vs. 84.7 ± 2.6% (r*Glut2* KO; *p* = 0.538, ns). For the fasted-state ^13^C_5_-glutamine tracer experiments, plasma M+5 enrichment was 44.4 ± 1.7% (Control) vs. 43.7 ± 1.8% (r*Glut2* KO; *p* = 0.795, ns). Taken together, these data confirm that differences in tissue isotopologue distributions across all tracer experiments reflect genuine metabolic differences rather than differential tracer delivery between r*Glut2* KO and control groups under either fed- or fasted-state conditions.

#### NMR analysis

Lyophilized polar extracts were reconstituted in D2O (>99.9%, Cambridge Isotope Laboratories, MA) containing 17.5 nmol d6-2,2-dimethyl-2-silapentane-5-sulfonate (DSS) as internal standard were analyzed by 1D ^1^H (PRESAT) and ^1^H(^13^C) -HSQC NMR on a Bruker 16.45 Avance III spectrometer equipped with a 1.7 mm HCN triple resonance cryoprobe. 1D ^1^H spectra were acquired at 15°C with 512 transients, 16384 data points, 12 ppm spectral width, an acquisition time of 2 s and a 6 s recycle time with weak irradiation on the residual HOD signal during the relaxation delay. The raw fids were apodized with 1 Hz exponential line broadening and zero filled to 131072 points prior to Fourier transformation. 1D ^1^H(^13^C)HSQC spectra were recorded with an acquisition time of 0.25 s with adiabatic decoupling and recycle time of 2 s over a spectral width of 12 ppm, with, 1024 transients. The HSQC spectra were then apodized with unshifted Gaussian function and 4 Hz exponential line broadening and zero filled to 16384 data points before Fourier transformation. Metabolites were assigned by comparison with in-house^41^ and public NMR databases. Metabolites and their ^13^C isotopomers were quantified using the MesReNova software (Mestrelab, Santiago de Compostela, Spain) by peak deconvolution. The peak areas of metabolites obtained were converted into nmoles by calibration against the peak intensity of DSS (17.5 nmoles) at 0 ppm for ^1^H spectra. Peak areas for each spectrum were corrected for the number of protons, calibrated against DSS to determine the absolute amount, and then normalized to tissue residue weight to obtain metabolite content as μmol/g residue. The fractional enrichment of site specific ^13^C (F) was calculated from the ^1^H spectra as:

F=A(C13)∕[A(C13)+A(C12)]

where A(^13^C) and A(^12^C) are the peak areas of the proton of interest directly attached to ^13^C and ^12^C, respectively.

#### *In vivo* studies

##### Hyperinsulinemic-hypoglycemic clamps

The glucose clamp study in mice was performed at UMass Metabolic Disease Research Center as described previously^42^ with some modifications. Blood glucose levels were clamped around 60 mg/dL using continuous insulin and glucose infusions. At the end of the clamp study, 2-[1-^14^C]deoxy-*d*-glucose was injected into the mice and tissues of interest were collected 45 min after the injection to determine tissue-specific insulin-stimulated glucose uptake.

##### Sympathetic nerve activity (SNA)

The adrenal and hepatic SNA were measured as described previously.^43^ After anesthesia, the nerve innervating the liver or adrenal gland was identified, dissected free, and placed on a bipolar 36-gauge platinum-iridium electrode (A-M Systems; Carlsborg, WA). The electrode was connected to a high-impedance probe (HIP-511; Grass Instruments Co., Quincy, MA), and the nerve signal was amplified 10^5^ times with a Grass P5 AC pre-amplifier and filtered at low and high-frequency cutoffs of 100 Hz and 1000 Hz, respectively. This nerve signal was directed to a speaker system and to an oscilloscope (54501 A, Hewlett–Packard Co., Palo Alto, CA) for auditory and visual monitoring of the nerve activity. The signal was then directed to a resetting voltage integrator (B600C, University of Iowa Bioengineering) that sums the total voltage output in units of 1 V × s before resetting to zero and counting the number of spikes per second. The final neurograms were continuously routed to a MacLab analogue–digital converter (8 S, AD Instruments Castle Hill, New South Wales, Australia) for permanent recording and data analysis on a Macintosh computer. The nerve activity was measured for 30 min.

##### Blood glucose measurement

V-9302 (75 mg/kg bodyweight, dissolved in 5% DMSO/95% sesame oil, %v/v, A1002473, Ambeed), dapagliflozin (10 mg/kg bodyweight, dissolved in PBS, A120381, Ambeed) and sotagliflozin (30 mg/kg bodyweight, dissolved in 5% DMSO/95% sesame oil, %v/v, A225622, Ambeed) were administered intraperitoneally to different cohorts of non-fasted C57BL/6J mice (000664, Jackson Laboratory). The mouse tail-end was nicked using a razor blade and the tail was gently massaged to obtain a drop of blood. A glucometer (Alpha Trak 3, Zoetis) was then used per the manufacturer’s instructions to measure the blood glucose levels at different times after the administration of drugs.

##### Indirect calorimetry

Energy expenditure and respiratory exchange ratio (RER) were measured by indirect calorimetry using metabolic cages over a 94-h recording period encompassing both light and dark cycles. Data were analyzed using CalR v1.3.^36^ Generalized linear models (GLM) with a Gaussian error distribution and identity link function were used for all statistical comparisons. Body mass was included as a covariate for energy expenditure, oxygen consumption, and carbon dioxide production, but not for RER, which is inherently mass-independent as it represents the ratio of CO_2_ produced to O_2_ consumed (VCO_2_/VO_2_). Group effects were considered significant at *p* < 0.05.

##### AAV-mediated renal Mpi knockout

To achieve kidney-specific knockout of *Mpi*, adult *Mpi^loxp/loxP^* mice (T016292, GemPharmatech) were anesthetized, and the kidneys were exposed via a midline laparotomy. AAV9-CMV-Cre (105537, Addgene), or AAV9-CMV-GFP as a control (105530, Addgene), in a 50 μL volume, was delivered via retrograde renal pelvis injection using a 33-gauge needle. To maximize transduction efficiency and minimize systemic leakage, the renal artery and vein were temporarily occluded with micro-vascular clamps for 15 min during and after the injection. Three weeks after AAV administration, mice were treated with dapagliflozin, and blood glucose levels were monitored as described above.

##### RT-qPCR

*Mpi* knockout efficiency in the kidney was quantified by RT-qPCR using *Hprt* as the internal reference gene for normalization. The following primers were used: *Mpi*: *5*′-CAGCACAGAGTCAAGATGACCC-3′ and 5′-GAAGGATGCTGGCAGAGTCTAG-3’; *Hprt*: *5*′-CTGGTGAAAAGGACCTCTCGAAG-3′ and 5′-CCAGTTTCACTAATGACACAAACG-3’

##### Western blotting

Western blotting was performed to measure renal MPI protein expression as described previously.^9,11^ Briefly, kidney protein lysates were prepared by homogenization in RIPA buffer supplemented with protease and phosphatase inhibitors, and protein concentration was determined by BCA assay (Cat#23225, Thermo Fisher Scientific). Equal amounts of protein were resolved by SDS-PAGE and transferred to PVDF membranes. Membranes were blocked in 5% non-fat dry milk in TBST for 1 h at room temperature, then incubated overnight at 4°C with primary antibodies: anti-MPI rabbit polyclonal antibody (Cat#14234-1-AP, Proteintech; 1:1000 dilution in TBST) and anti-Vinculin rabbit antibody (Cat#26520-1-AP, Proteintech; 1:2000 dilution in TBST) as loading control. Blots were cut horizontally above the appropriate molecular weight to allow simultaneous probing of the protein of interest and loading control on the same membrane, or membranes were stripped and reprobed. HRP-conjugated anti-rabbit IgG secondary antibody (Cat#31460, Pierce/Thermo Scientific; 1:5000 dilution in TBST) was used for detection. Membranes were developed using ECL substrate (Cat#34095, Thermo Fisher Scientific) and imaged on a ChemiDoc XRS+ system (Bio-Rad). Band intensities were quantified using Image Lab v6.1 software and normalised to Vinculin.

##### Cytokine array

We used a mouse cytokine array (ARY028, R&D systems) to analyze serum of r*Glut2* KO mice and their littermate controls per the manufacturer’s instructions. We collected 50 μL serum from each mouse. Then, the serum of three mice in each group was pooled into 2mL microtubes separately for the control and experimental groups. The array membranes were developed using chemiluminescent detection reagents, imaged via ChemiDoc XRS+ (Bio-Rad Laboratories) and analyzed through Image Lab 6.1 software. Data are presented as exploratory due to pooled serum design (three mice per pool), which precludes inference of individual-level variability.

##### Single-nucleus multiome RNA-seq and ATAC-seq

For multiomic gene analysis, we isolated nuclei from three flash-frozen mouse kidney samples per group and constructed single nucleus gene expression libraries and single nucleus transposase-accessible chromatin regions (ATAC) libraries on the 10x genomics chromium system using the 10x chromium multiome kit (Singulomics Corporation). Each library was sequenced with ~200 million pair end reads (PE150) on Illumina NovaSeq. Using the 10x solution, we assayed for ATAC alongside gene expression (RNA-seq) for combined epigenomic and transcriptomic analysis from the same single nucleus. For each sample, we applied Seurat v5.1.0 and Signac v 1.13.0 to create a multiomic Seurat object with paired gene expression and DNA accessibility profiles for each sample. The sequencing reads were analyzed with mouse reference genome mm10 (2020-A) using the Cell Ranger ARC 2.0.2 count function. The transcription factor activity was predicted based on the presence of binding motifs within differential accessibility chromatin regions using chromVAR and the positional weight matrix (https://genome.ucsc.edu/goldenPath/help/hgRegMotifHelp.html) was obtained from the JASPAR2020 database (https://jaspar2020.genereg.net/). For each cell type, we identified differential accessibility *cis*-regulatory elements at ATAC-seq level and calculated marker genes at the RNA level. Additionally, we generated a gene activity matrix inferred from ATAC-seq by using the GeneActivity function using Signac.

##### Reduced-representation bisulfite sequencing (RRBS)

Reduced-representation bisulfite sequencing (RRBS) was performed at Zymo Research, Irvine, CA. 500 ng of starting input genomic DNA was digested with 30 units of MspI (R0106, NEB). Fragments were ligated to pre-annealed adapters containing 5′-methyl-cytosine according to Illumina’s specified guidelines. Adaptor-ligated fragments ≥50 bp in size were recovered using the DNA Clean & Concentrator-5 (D4003, Zymo Research). The fragments were then bisulfite-treated using the EZ DNA Methylation-Lightning Kit (D5030, Zymo Research). Preparative-scale PCR was performed and the resulting products were purified with DNA Clean & Concentrator-5 (D4003, Zymo Research). Library quality control was performed on the Agilent 4200 TapeStation. Libraries were sequenced on an Illumina NovaSeq X platform (150 bp PE reads). The sequence reads from RRBS libraries (non-directional) were identified using standard Illumina base calling software. To process RRBS data, the trimmed reads were aligned to the mouse genome assembly using Bismark. Methylation ratios for each cytosine in CpG context were called using MethylDackel. The methylation level of each sampled cytosine was estimated as the number of reads reporting a C, divided by the total number of reads reporting a C or T. Read depths per cytosine in the genome as well as in different genomic regions (e.g., gene body, promoter, CpG island, etc. based on available annotations) were calculated and tabulated using scripts.

### QUANTIFICATION AND STATISTICAL ANALYSIS

*In vivo* data are shown as mean ± SEM. Results were analyzed by two-tailed Student’s unpaired *t* test, multiple comparison *t* test, or repeated measures two-way ANOVA followed by a Holm-Sidak or Bonferroni post hoc multiple comparison test when appropriate. All analyses were performed using Prism version 10.2.3 (GraphPad, USA) and differences were considered statistically significant at *p* < 0.05.

For multiomic single nucleus RNA and ATAC sequencing, the analysis of each cell type was carried out using the Wilcoxon Rank-Sum Test, implemented through the Seurat FindAllMarkers function. The differentially expressed genes and accessibility were considered statistically significant at false discovery rate (FDR) adjusted *p* < 0.05. The pathway analysis for cross-condition comparisons involves evaluating overexpressed and underexpressed genes within each cell type using Gene Set Enrichment Analysis (GSEA). GSEA considers the magnitude of differential expression for each gene by applying gene-specific weights. The pathways selected for analysis are obtained from the Molecular Signatures Database through the R package msigdbr.

To compare RRBS results between the groups, a statistical analysis was performed to identify, annotate, and visualize differential methylated sites and regions using differential shrinkage sequencing (DSS) package (software Bioconductor 3.22). Differentially methylated cytosines (DMCs) and differentially methylated regions (DMRs) were detected using DSS, which by default conducted the Wald test and the Benjamini-Hochberg P-value adjustment. Significant DMCs and DMRs have FDR ≤0.05 and absolute methylation difference ≥0.1.

## Supplementary Material

1

2

3

4

5

Supplemental information can be found online at https://doi.org/10.1016/j.celrep.2026.117804.

## Figures and Tables

**Table:** KEY RESOURCES TABLE

REAGENT or RESOURCE	SOURCE	IDENTIFIER
Antibodies		
Anti-MPI rabbit polyclonal antibody	Proteintech	Cat# 14234-1-AP; RRID:AB_2146302
Anti-Vinculin rabbit antibody	Proteintech	Cat# 26520-1-AP
HRP-conjugated anti-rabbit IgG	Pierce, Thermo Scientific	Cat# 31460; RRID:AB_228341
Bacterial and virus strains		
AAV9-CMV-Cre	Addgene	Addgene AAV9; Cat#105537
AAV9-CMV-GFP (control vector)	Addgene	Addgene AAV9; Cat#105530
Biological samples		
Mouse kidney, liver, soleus muscle, trunk blood/plasma, urine (flash-frozen)	This paper	N/A
Chemicals, peptides, and recombinant proteins		
^13^C_6_-glucose (U-^13^C_6_, 99%)	Cambridge Isotope Laboratories	CLM-1396-0.25
^13^C_6_-mannose (U-^13^C_6_, 99%)	Cambridge Isotope Laboratories	CLM-6567-0.25
^13^C_5_-glutamine (U-^13^C_5_, 99%)	Cambridge Isotope Laboratories	CLM-1822-H-0.25
2-[1-^14^C]deoxy-D-glucose	PerkinElmer	Cat#NEC495A050UC
V-9302 (ASCT2/SLC1A5 inhibitor)	Ambeed	Cat#A1002473; CAS: 1467176-62-0
Dapagliflozin (SGLT2 inhibitor)	Ambeed	Cat#A120381; CAS: 461432-26-8
Sotagliflozin (dual SGLT1/SGLT2 inhibitor)	Ambeed	Cat#A225622; CAS: 1018899-04-1
Tamoxifen	Sigma-Aldrich	Cat#T5648; CAS: 10540-29-1
D_2_O (>99.9% D)	Cambridge Isotope Laboratories	DLM-4-100
EZ DNA Methylation-Lightning Kit	Zymo Research	Cat#D5030
DNA Clean & Concentrator-5	Zymo Research	Cat#D4003
Critical commercial assays		
Mouse Cytokine Array Panel A	R&D Systems	Cat#ARY028
10x Chromium Multiome ATAC & Gene Expression Kit	10x Genomics	Cat#PN-1000285
Pierce BCA Protein Assay Kit	Thermo Fisher Scientific	Cat#23225
Deposited data		
Single-nucleus multiome (RNA-seq + ATAC-seq) and RRBS data — SuperSeries (r*Glut2* KO vs. control kidney)	This paper	GEO: GSE332903
Single-nucleus multiome RNA-seq and ATAC-seq — SubSeries	This paper	GEO: GSE331205
Reduced-representation bisulfite sequencing (RRBS) — SubSeries	This paper	GEO: GSE332732
Differentially expressed genes per kidney cell type	This paper	Data S2
Differentially accessible chromatin regions per kidney cell type	This paper	Data S3
Differentially methylated cytosines and regions (RRBS)	This paper	Data S4
Experimental models: Cell lines		
N/A — no cell lines used	N/A	N/A
Experimental models: Organisms/strains		
Mouse: tamoxifen-inducible kidney-specific *Glut2* KO; C57BL/6J background	de Souza Cordeiro et al.^2^	N/A
Mouse: C57BL/6J (wildtype)	The Jackson Laboratory	Cat#000664; RRID: IMSR_JAX:000664
Mouse: *Mpi*flox/flox (for AAV-mediated renal Mpi KO)	GemPharmatech	Cat#T016292
Oligonucleotides		
RT-qPCR Forward primer: *Mpi*: 5′-CAGCACAGAGTCAAGATGACCC-3′	This paper	N/A
RT-qPCR Reverse primer: *Mpi*: 5′-GAAGGATGCTGGCAGAGTCTAG-3′	This paper	N/A
RT-qPCR Forward primer: *Hprt*: 5′-CTGGTGAAAAGGACCTCTCGAAG-3′	This paper	N/A
RT-qPCR Reverse primer: *Hprt*: 5′-CCAGTTTCACTAATGACACAAACG-3′	This paper	N/A
Recombinant DNA		
pAAV-CMV-Cre	Addgene	Addgene Plasmid #105537
pAAV-CMV-GFP	Addgene	Addgene Plasmid #105530
Software and algorithms		
GraphPad Prism v10.2.3	GraphPad Software	https://www.graphpad.com; RRID: SCR_002798
Seurat v5.1.0	Open source software	https://satijalab.org/seurat/; RRID: SCR_016341
Signac v1.13.0	Open source software	https://stuartlab.org/signac/
Cell Ranger ARC v2.0.2	10x Genomics	https://www.10xgenomics.com; RRID: SCR_023221
chromVAR	Open source software	https://bioconductor.org/packages/chromVAR; RRID: SCR_017206
JASPAR2020 database	Open source software	https://jaspar2020.genereg.net/
Bismark v0.22	Open source software	https://www.bioinformatics.babraham.ac.uk/projects/bismark/; RRID: SCR_005604
MethylDackel	Open source software	https://github.com/dpryan79/MethylDackel
DSS (Bioconductor v3.22)	Open source software	https://bioconductor.org/packages/DSS
msigdbr R package	Open source software	https://cran.r-project.org/package=msigdbr
TraceFinder v4.1	Thermo Fisher Scientific	N/A
MestReNova v14	Mestrelab Research	https://mestrelab.com; RRID: SCR_017780
Image Lab v6.1	Bio-Rad Laboratories	https://www.bio-rad.com; RRID: SCR_014210
IsoCor	Millard et al.^35^	https://github.com/MetaSys-LISBP/IsoCor
CalR v1.3	Mina et al.^36^	https://calrapp.org; RRID: SCR_021038
Other		
Dionex IonPac AG11-HC/AS11-HC ion chromatography columns	Thermo Scientific	N/A
Orbitrap Fusion Tribrid mass spectrometer	Thermo Fisher Scientific	N/A
Bruker Avance III 16.45 T NMR spectrometer with 1.7 mm HCN cryoprobe	Bruker	N/A
Agilent 4200 TapeStation	Agilent Technologies	N/A
Illumina NovaSeq X (150 bp PE)	Illumina	N/A
ChemiDoc XRS+ imaging system	Bio-Rad Laboratories	N/A
AlphaTrak 3 glucometer	Zoetis	N/A
GC-TOF-MS untargeted metabolomics service	West Coast Metabolomics Center, UC Davis	N/A

## Highlights

- Renal glycosuria increases mannose metabolism in the kidney
- Renal glycosuria induces systemic glucose-sparing adaptations
- Glutamine metabolism supports renal and systemic glucose compensation
- Targeting mannose and glutamine may improve SGLT2-inhibitor efficacy
