# Supplementary material for: Renal glycosuria triggers a coordinated mannose and glutamine metabolic program to maintain glucose homeostasis
Source: Cell Rep. Author manuscript; Available in PMC 2026 Sep 15. (PMC13576360; doi:10.1016/j.celrep.2026.117804)
Supplement: Supplementary file 1 [file NIHMS2206917-supplement-1.pdf]

Cell Reports, Volume *45*

Supplemental information

Renal glycosuria triggers a coordinated mannose and glutamine metabolic program to maintain glucose homeostasis

Nadia Rashid, Moses Otunla, Nazmul Hasan, Michael J. Hodges, Heba H. Qaissi, Tumininu S. Faniyan, Pontiana Ritika Clement, Penghui Lin, Mohamed M.Y. Kaddah, Teresa A. Cassel, Donald A. Morgan, Kamal Rahmouni, and Kavaljit H. Chhabra

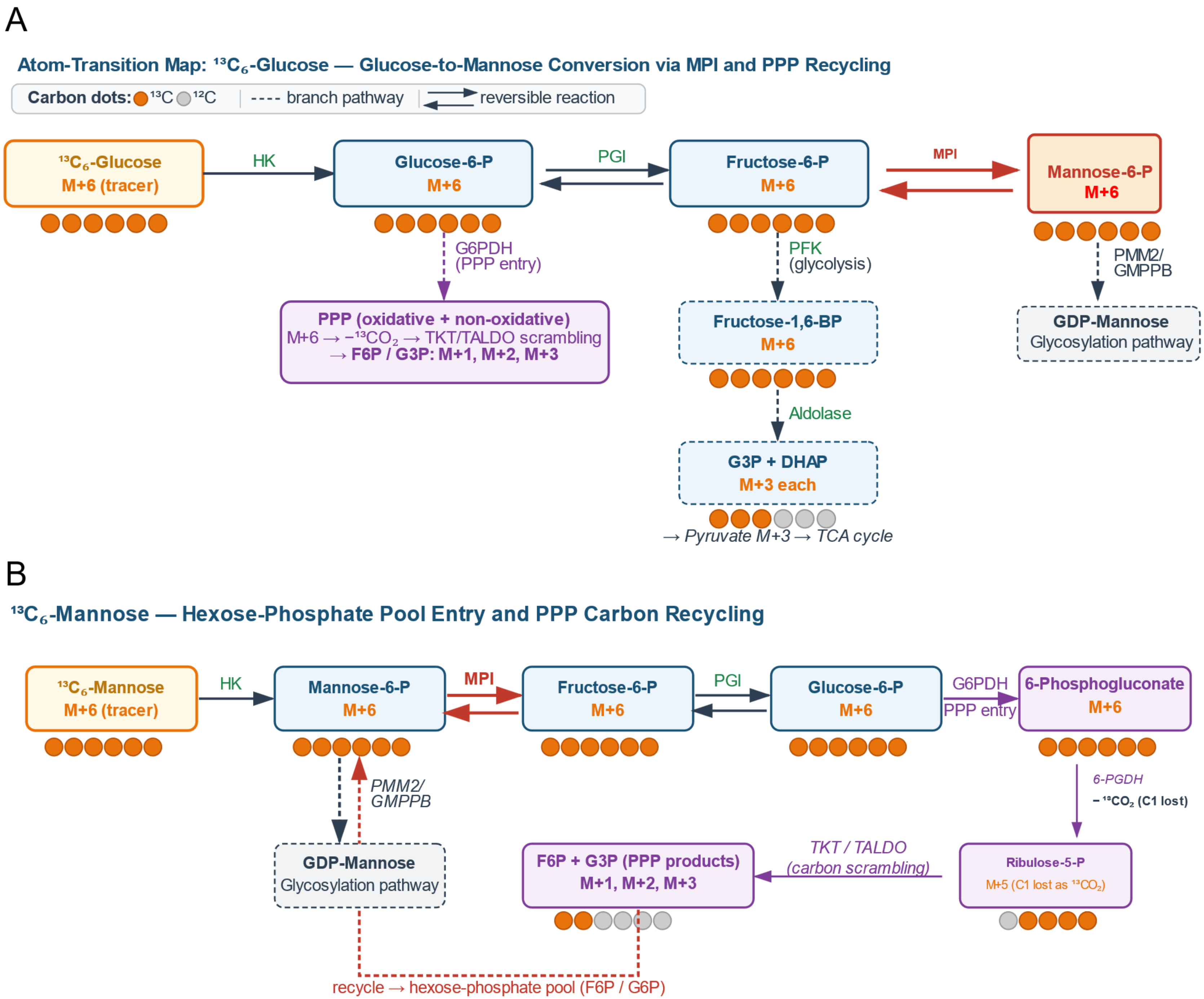

A
Atom-Transition Map: ¹³C₆-Glucose — Glucose-to-Mannose Conversion via MPI and PPP Recycling
Carbon dots: ¹³C ¹²C
---- branch pathway
reversible reaction
¹³C₆-Glucose
M+6 (tracer)
HK
Glucose-6-P
M+6
PGI
Fructose-6-P
M+6
MPI
Mannose-6-P
M+6
G6PDH
(PPP entry)
PFK
(glycolysis)
PMM2/
GMPPB
PPP (oxidative + non-oxidative)
M+6 → −¹³CO₂ → TKT/TALDO scrambling
→ F6P / G3P: M+1, M+2, M+3
Fructose-1,6-BP
M+6
GDP-Mannose
Glycosylation pathway
Aldolase
G3P + DHAP
M+3 each
→ Pyruvate M+3 → TCA cycle
B
¹³C₆-Mannose — Hexose-Phosphate Pool Entry and PPP Carbon Recycling
¹³C₆-Mannose
M+6 (tracer)
HK
Mannose-6-P
M+6
MPI
Fructose-6-P
M+6
PGI
Glucose-6-P
M+6
G6PDH
PPP entry
6-Phosphogluconate
M+6
PMM2/
GMPPB
6-PGDH
− ¹³CO₂ (C1 lost)
GDP-Mannose
Glycosylation pathway
F6P + G3P (PPP products)
M+1, M+2, M+3
TKT / TALDO
(carbon scrambling)
Ribulose-5-P
M+5 (C1 lost as ¹³CO₂)
recycle → hexose-phosphate pool (F6P / G6P)

Figure S1. Atom-transition maps illustrating ^{13}C carbon fate through the hexose-phosphate pool, related to Figures 1-3. These maps illustrate expected isotopologue distributions arising from $^{13}C_6$-glucose and $^{13}C_6$-mannose tracers, including contributions from glycolysis and pentose phosphate pathway (PPP) carbon recycling. Orange dots indicate ^{13}C-labeled carbons; gray dots indicate ^{12}C. M+N denotes isotopologue mass. Dashed arrows represent branch pathways, and bidirectional arrows indicate reversible reactions. These pathways explain the mixed isotopologue patterns (M+1–M+3 and M+6) observed experimentally in the hexose-phosphate pool. **(A)** $^{13}C_6$-Glucose tracer. $^{13}C_6$-glucose (M+6) is phosphorylated by hexokinase (HK) to glucose-6-phosphate (G6P, M+6), which can enter three routes: (1) phosphoglucose isomerase (PGI) to fructose-6-phosphate (F6P), followed by mannose phosphate isomerase (MPI) to mannose-6-phosphate (M6P), illustrating that glucose-to-mannose carbon flux is biochemically feasible and that M+6 M6P is most consistent with derivation from $^{13}C_6$-glucose via this pathway; (2) entry into the PPP via glucose-6-phosphate dehydrogenase (G6PDH), followed by transketolase (TKT) and transaldolase (TALDO)-mediated carbon rearrangements, generating M+1, M+2, and M+3 isotopologues that recycle into the hexose-phosphate pool; and (3) glycolytic flux through phosphofructokinase (PFK) to generate triose phosphates and M+3 pyruvate, which feeds into the TCA cycle. **(B)** $^{13}C_6$-Mannose tracer. $^{13}C_6$-mannose (M+6) enters the hexose-phosphate pool through HK and MPI, producing predominantly M+6-labeled mannose-6-phosphate, fructose-6-phosphate, and glucose-6-phosphate. A fraction enters the PPP, where decarboxylation yields ribulose-5-phosphate (M+5) and subsequent carbon rearrangements generate mixed lower-mass isotopologues (M+1–M+3). These labeled species coexist with M+6 intermediates in the hexose-phosphate pool and are consistent with PPP-mediated carbon recycling.

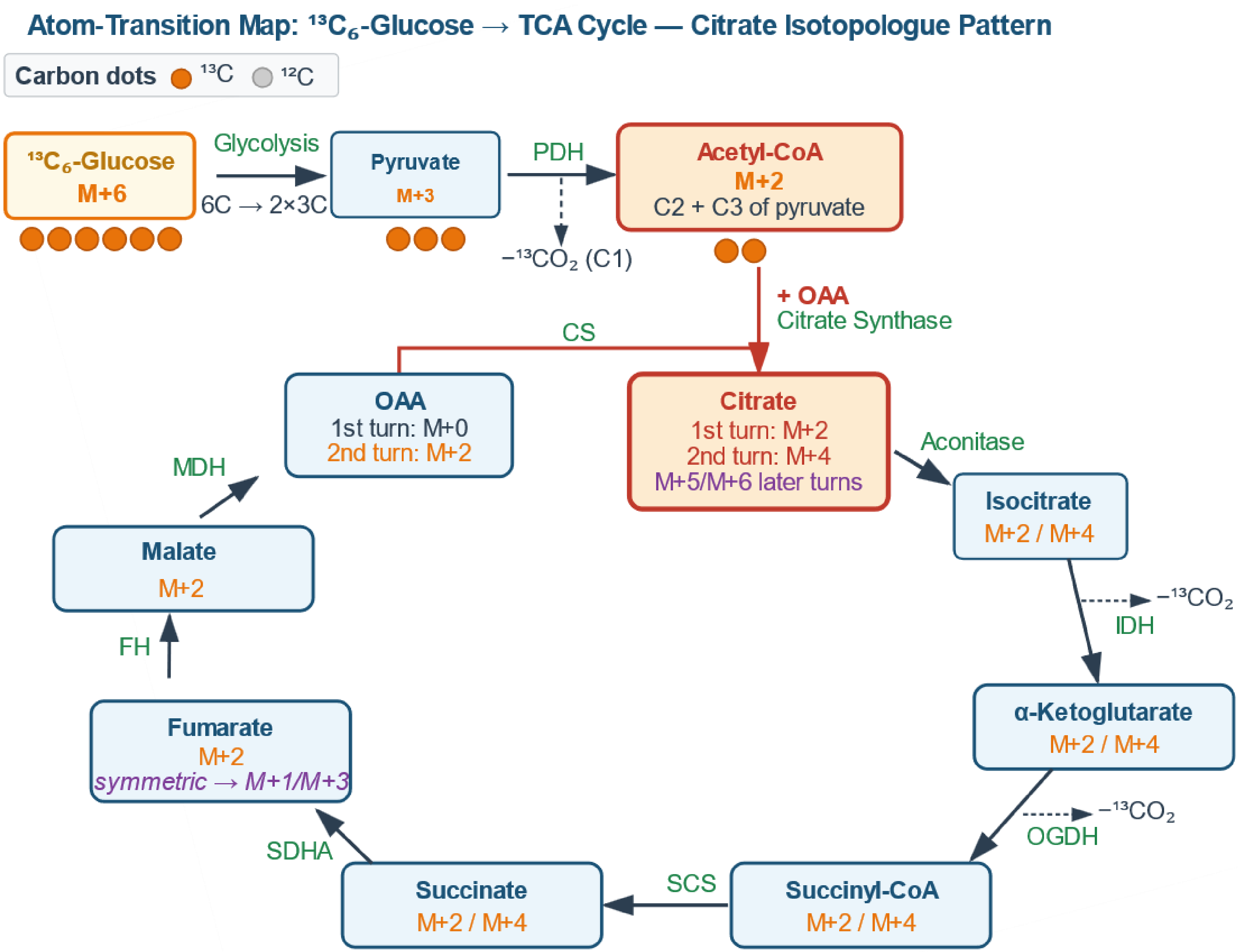

Atom-Transition Map: $^{13}C_6$-Glucose → TCA Cycle — Citrate Isotopologue Pattern
Carbon dots ^{13}C ^{12}C
$^{13}C_6$-Glucose M+6
Glycolysis
6C → 2×3C
Pyruvate M+3
PDH
$-^{13}CO_2$ (C1)
Acetyl-CoA M+2 C2 + C3 of pyruvate
+ OAA
Citrate Synthase
CS
OAA 1st turn: M+0 2nd turn: M+2
Citrate 1st turn: M+2 2nd turn: M+4 M+5/M+6 later turns
Aconitase
Isocitrate M+2 / M+4
$-^{13}CO_2$
IDH
α-Ketoglutarate M+2 / M+4
$-^{13}CO_2$
OGDH
Succinyl-CoA M+2 / M+4
SCS
Succinate M+2 / M+4
SDHA
Fumarate M+2 symmetric → M+1/M+3
FH
Malate M+2
MDH

Figure S2. Atom-transition map illustrating ^{13}C carbon fate through the TCA cycle from $^{13}C_6$-glucose, related to Figures 4 and 5. This map illustrates how $^{13}C_6$-glucose-derived carbon propagates through glycolysis and the TCA cycle, and how sequential turns generate characteristic isotopologue distributions. Orange dots indicate ^{13}C-labeled carbons; gray dots indicate ^{12}C. M+N denotes isotopologue mass. Carbon dots are shown for glycolytic intermediates only; TCA cycle intermediates are labeled by isotopologue class. $^{13}C_6$-Glucose is metabolized through glycolysis to pyruvate (M+3). Pyruvate dehydrogenase (PDH) releases C1 as $^{13}CO_2$, yielding acetyl-CoA (M+2). Acetyl-CoA condenses with oxaloacetate (OAA) via citrate synthase to enter the TCA cycle, progressing through aconitase, isocitrate dehydrogenase, α-ketoglutarate dehydrogenase, succinyl-CoA synthetase, succinate dehydrogenase, fumarase, and malate dehydrogenase. The expected citrate isotopologue pattern evolves across TCA turns: first turn—M+2 citrate (acetyl-CoA M+2 + OAA M+0); second turn—M+4 citrate; subsequent turns—M+5 and M+6 species as labeled carbon accumulates in OAA. The presence of multiple citrate isotopologues (M+2, M+4, M+5, and M+6) is consistent with multi-turn TCA cycling under in vivo conditions. Odd isotopologues (M+1, M+3) are commonly attributed to carbon position scrambling at symmetric intermediates such as succinate and fumarate, which redistribute labeling in subsequent cycles. These labeling patterns explain the distributed isotopologue enrichment observed experimentally and the absence of a single dominant M+2 citrate peak. All isotope tracing data were corrected for natural isotope abundance (NIA). Plasma ^{13}C enrichment (~36%) did not differ significantly between groups, supporting that tissue isotopologue differences reflect metabolic processes rather than differential tracer delivery.

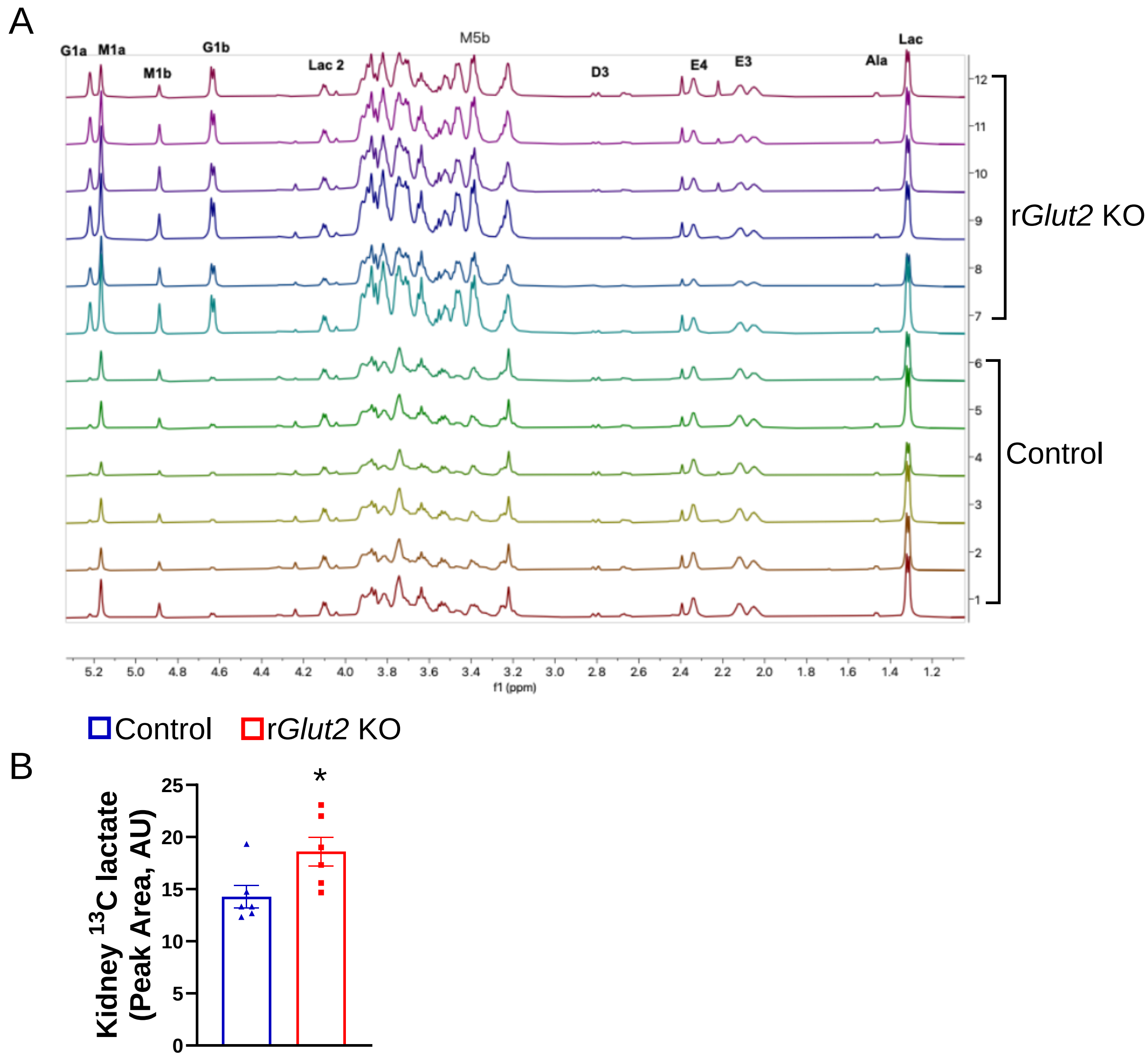
A
G1a
M1a
M1b
G1b
Lac 2
M5b
D3
E4
E3
Ala
Lac
rGlut2 KO
Control
f1 (ppm)
B
Control
rGlut2 KO
Kidney 13C lactate (Peak Area, AU)
*

Figure S3. NMR analyses of kidney samples collected from 14-week-old male renal glucose transporter 2 knockout (r*Glut2* KO) mice and their control littermates 60 minutes after $^{13}C_6$-mannose administration, related to Figures 1 and 2. **(A)** Representative NMR spectra. G, ^{13}C-glucose; M, ^{13}C-mannose; Lac, ^{13}C-lactate. Numbers indicate the carbon position of ^{13}C labeling, and letter annotations (e.g., a, b) denote distinct spin states of the corresponding nuclei. The x-axis represents parts per million. **(B)** ^{13}C-lactate in the kidney, consistent with increased incorporation of mannose-derived carbon into lactate. Peak area normalized to d_6-2,2-dimethyl-2-silapentane-5-sulfonate (DSS), arbitrary units (AU). *$p<0.05$, two-tailed unpaired t-test. N=6. Data are represented as mean ± SEM.

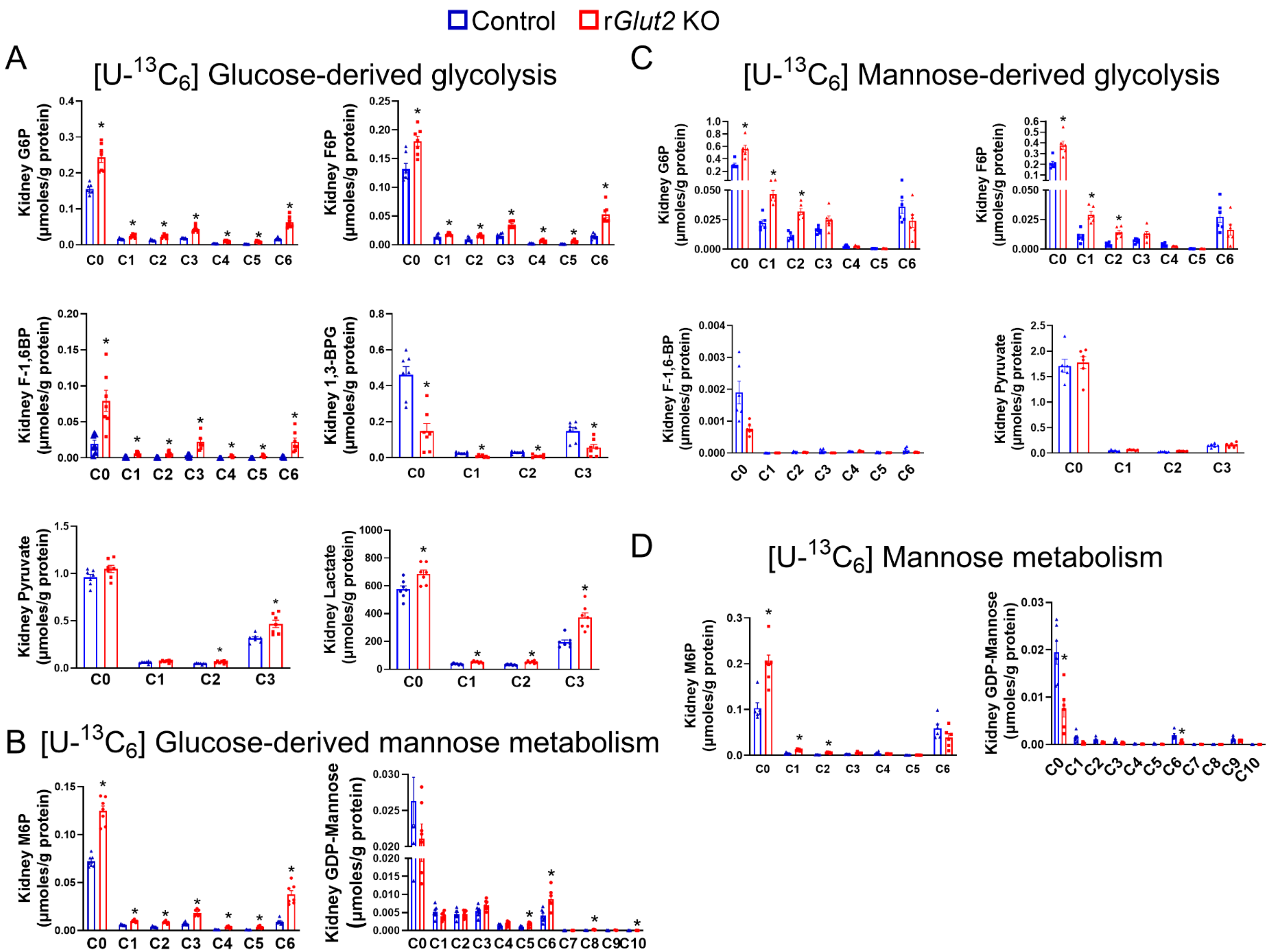
Control
rGlut2 KO
A
[U-13C6] Glucose-derived glycolysis
Kidney G6P (µmoles/g protein)
Kidney F6P (µmoles/g protein)
Kidney F-1,6BP (µmoles/g protein)
Kidney 1,3-BPG (µmoles/g protein)
Kidney Pyruvate (µmoles/g protein)
Kidney Lactate (µmoles/g protein)
B [U-13C6] Glucose-derived mannose metabolism
Kidney M6P (µmoles/g protein)
Kidney GDP-Mannose (µmoles/g protein)
C
[U-13C6] Mannose-derived glycolysis
Kidney G6P (µmoles/g protein)
Kidney F6P (µmoles/g protein)
Kidney F-1,6-BP (µmoles/g protein)
Kidney Pyruvate (µmoles/g protein)
D
[U-13C6] Mannose metabolism
Kidney M6P (µmoles/g protein)
Kidney GDP-Mannose (µmoles/g protein)

Figure S4. Stable isotope-resolved metabolomics analysis (by Ion Chromatography–Ultra-High Resolution Fourier Transform Mass Spectrometry) of kidney glucose and mannose metabolism in 14-week-old male renal glucose transporter 2 knockout (r*Glut2* KO) mice and their control littermates 60 minutes after $^{13}C_6$-glucose **(A, B)** or $^{13}C_6$-mannose **(C, D)** administration, related to Figures 1 and 2. **(A)** $^{13}C_6$-glucose labeling of glucose-6-phosphate (G6P), fructose-6-phosphate (F6P), fructose-1,6-bisphosphate (F1,6BP), pyruvate, and lactate was increased, whereas labeling of 1,3-bisphosphoglycerate (1,3-BPG) was decreased in r*Glut2* KO kidneys, consistent with altered labeling patterns across glycolytic intermediates and suggestive of bidirectional flux or carbon cycling within upper glycolysis. **(B)** $^{13}C_6$-glucose incorporation into mannose-6-phosphate (M6P) and GDP-mannose was increased in r*Glut2* KO kidneys, consistent with increased routing of hexose carbons into the mannose/M6P pathway. **(C)** $^{13}C_6$-mannose incorporation into hexose-phosphate and glycolytic intermediates (G6P and F6P) was increased, with minimal change in fructose-1,6-bisphosphate and pyruvate, consistent with increased mannose entry into the hexose-phosphate pool during glycosuria, with limited progression into downstream glycolytic steps. **(D)** $^{13}C_6$-mannose incorporation into M6P was increased whereas incorporation into GDP-mannose was decreased in r*Glut2* KO kidneys, consistent with enhanced mannose pathway engagement and accumulation of M6P for gluconeogenic or upstream metabolic pathways rather than glycosylation. Data are shown as absolute metabolite abundance (µmol/g protein) for each isotopologue. The x-axis represents isotopologue distribution (C0–Cn), corresponding to the number of ^{13}C atoms incorporated into each metabolite. N=7, *$p<0.05$, multiple comparison unpaired t-test corrected using Holm-Sidak method. Data are represented as mean ± SEM.

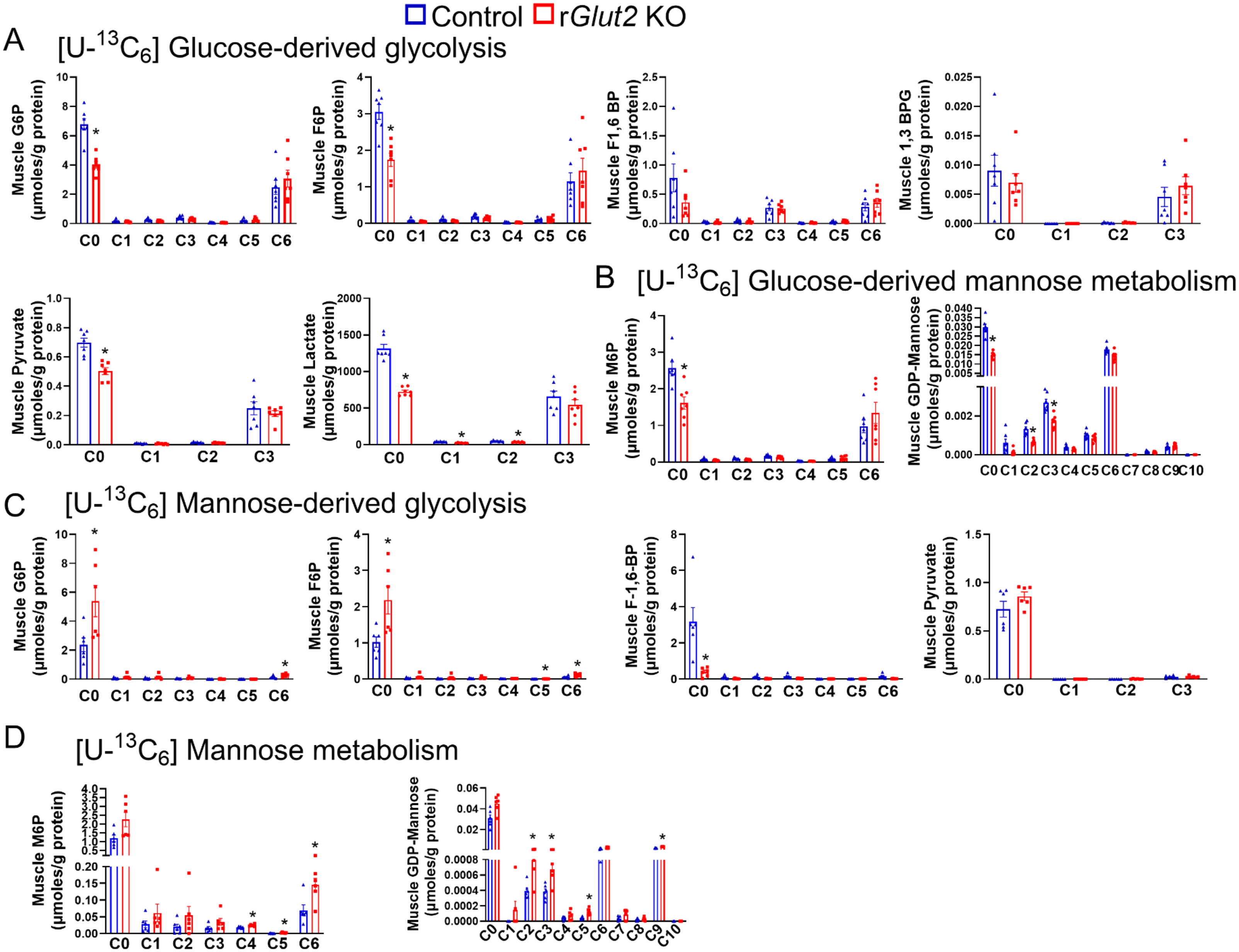

Control rGlut2 KO
A [U-13C6] Glucose-derived glycolysis
Muscle G6P (µmoles/g protein)
Muscle F6P (µmoles/g protein)
Muscle F1,6 BP (µmoles/g protein)
Muscle 1,3 BPG (µmoles/g protein)
Muscle Pyruvate (µmoles/g protein)
Muscle Lactate (µmoles/g protein)
B [U-13C6] Glucose-derived mannose metabolism
Muscle M6P (µmoles/g protein)
Muscle GDP-Mannose (µmoles/g protein)
C [U-13C6] Mannose-derived glycolysis
Muscle G6P (µmoles/g protein)
Muscle F6P (µmoles/g protein)
Muscle F-1,6-BP (µmoles/g protein)
Muscle Pyruvate (µmoles/g protein)
D [U-13C6] Mannose metabolism
Muscle M6P (µmoles/g protein)
Muscle GDP-Mannose (µmoles/g protein)
C0 C1 C2 C3 C4 C5 C6 C7 C8 C9 C10

Figure S5. Stable isotope-resolved metabolomics analysis (by Ion Chromatography–Ultra-High Resolution Fourier Transform Mass Spectrometry) of soleus muscle glucose and mannose metabolism in 14-week-old male r*Glut2* KO mice and their control littermates 60 minutes after $^{13}C_6$-glucose or $^{13}C_6$-mannose administration, related to Figure 3. **(A)** $^{13}C_6$-glucose labeling of glucose-6-phosphate (G6P), fructose-6-phosphate (F6P), fructose-1,6-bisphosphate (F1,6BP), 1,3-bisphosphoglycerate (1,3-BPG), pyruvate, and lactate in r*Glut2* KO mice and controls. The unlabeled fraction (C0) of these glycolytic intermediates was lower in r*Glut2* KO muscle, consistent with altered glucose utilization and labeling patterns in skeletal muscle. **(B)** $^{13}C_6$-glucose incorporation into muscle mannose-6-phosphate (M6P) and GDP-mannose was reduced in r*Glut2* KO mice, consistent with reduced pool size despite increased fractional enrichment (Figure 3B). **(C)** $^{13}C_6$-mannose labeling of hexose-phosphate and glycolytic intermediates (G6P and F6P) and **(D)** $^{13}C_6$-mannose labeling of mannose metabolites (M6P and GDP-mannose) was increased in r*Glut2* KO mice, whereas fructose-1,6-bisphosphate was decreased and pyruvate was unchanged. These patterns are consistent with increased contribution of mannose-derived carbon to glycolytic intermediates in skeletal muscle in the context of reduced glucose utilization. Data are shown as absolute metabolite abundance (µmol/g protein) for each isotopologue. The x-axis represents isotopologue distribution (C0–Cn), corresponding to the number of ^{13}C atoms incorporated into each metabolite. N=7, *$p<0.05$, multiple comparison unpaired t-test corrected using Holm-Sidak method. Data are represented as mean ± SEM.

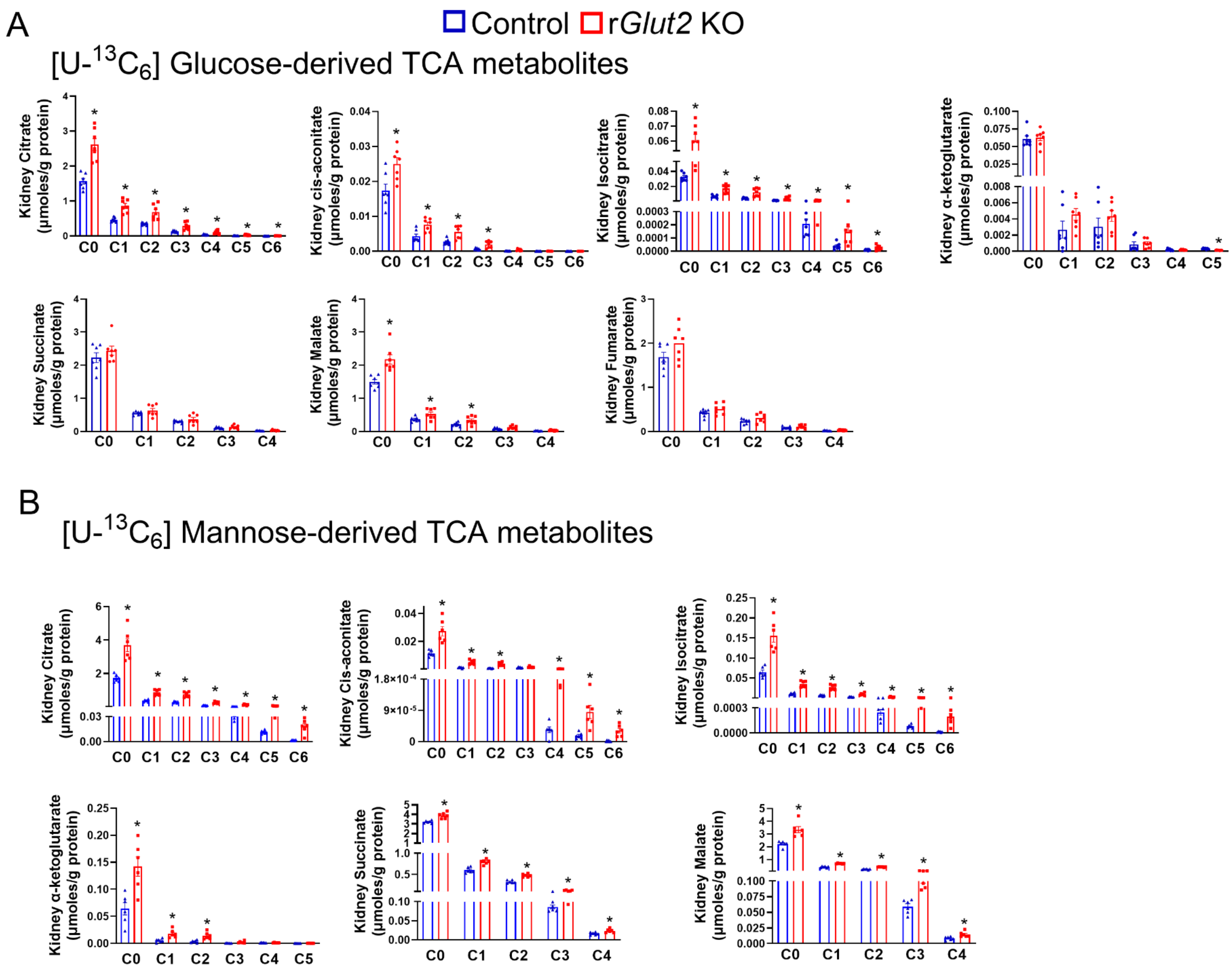

A
Control rGlut2 KO
[U-13C6] Glucose-derived TCA metabolites
Kidney Citrate (µmoles/g protein)
Kidney cis-aconitate (µmoles/g protein)
Kidney Isocitrate (µmoles/g protein)
Kidney α-ketoglutarate (µmoles/g protein)
Kidney Succinate (µmoles/g protein)
Kidney Malate (µmoles/g protein)
Kidney Fumarate (µmoles/g protein)
C0 C1 C2 C3 C4 C5 C6
B
[U-13C6] Mannose-derived TCA metabolites
Kidney Citrate (µmoles/g protein)
Kidney Cis-aconitate (µmoles/g protein)
Kidney Isocitrate (µmoles/g protein)
Kidney α-ketoglutarate (µmoles/g protein)
Kidney Succinate (µmoles/g protein)
Kidney Malate (µmoles/g protein)
C0 C1 C2 C3 C4 C5 C6

Figure S6. Stable isotope-resolved metabolomics analysis (by Ion Chromatography–Ultra-High Resolution Fourier Transform Mass Spectrometry) of kidney tricarboxylic acid (TCA) cycle metabolites in 14-week-old male r*Glut2* KO mice and their control littermates 60 minutes after $^{13}C_6$-glucose or $^{13}C_6$-mannose administration, related to Figure 4. **(A)** $^{13}C_6$-glucose incorporation into citrate, cis-aconitate, isocitrate, α-ketoglutarate, succinate, malate, and fumarate in r*Glut2* KO kidneys and controls. Citrate, cis-aconitate, isocitrate, and malate were increased, whereas succinate and fumarate showed little or no change, consistent with differential labeling across upstream and downstream TCA intermediates. α-Ketoglutarate was modestly reduced, consistent with reduced pool size despite increased fractional enrichment (Figure 4A). **(B)** $^{13}C_6$-mannose incorporation into citrate, cis-aconitate, isocitrate, α-ketoglutarate, succinate, and malate was increased in r*Glut2* KO kidneys, consistent with increased contribution of mannose-derived carbon to TCA cycle intermediates during glycosuria. Data are shown as absolute metabolite abundance (µmol/g protein) for each isotopologue. The x-axis represents isotopologue distribution (C0–Cn), corresponding to the number of ^{13}C atoms incorporated into each metabolite. N=7, *$p<0.05$, multiple comparison unpaired t-test corrected using Holm-Sidak method. Data are represented as mean ± SEM.

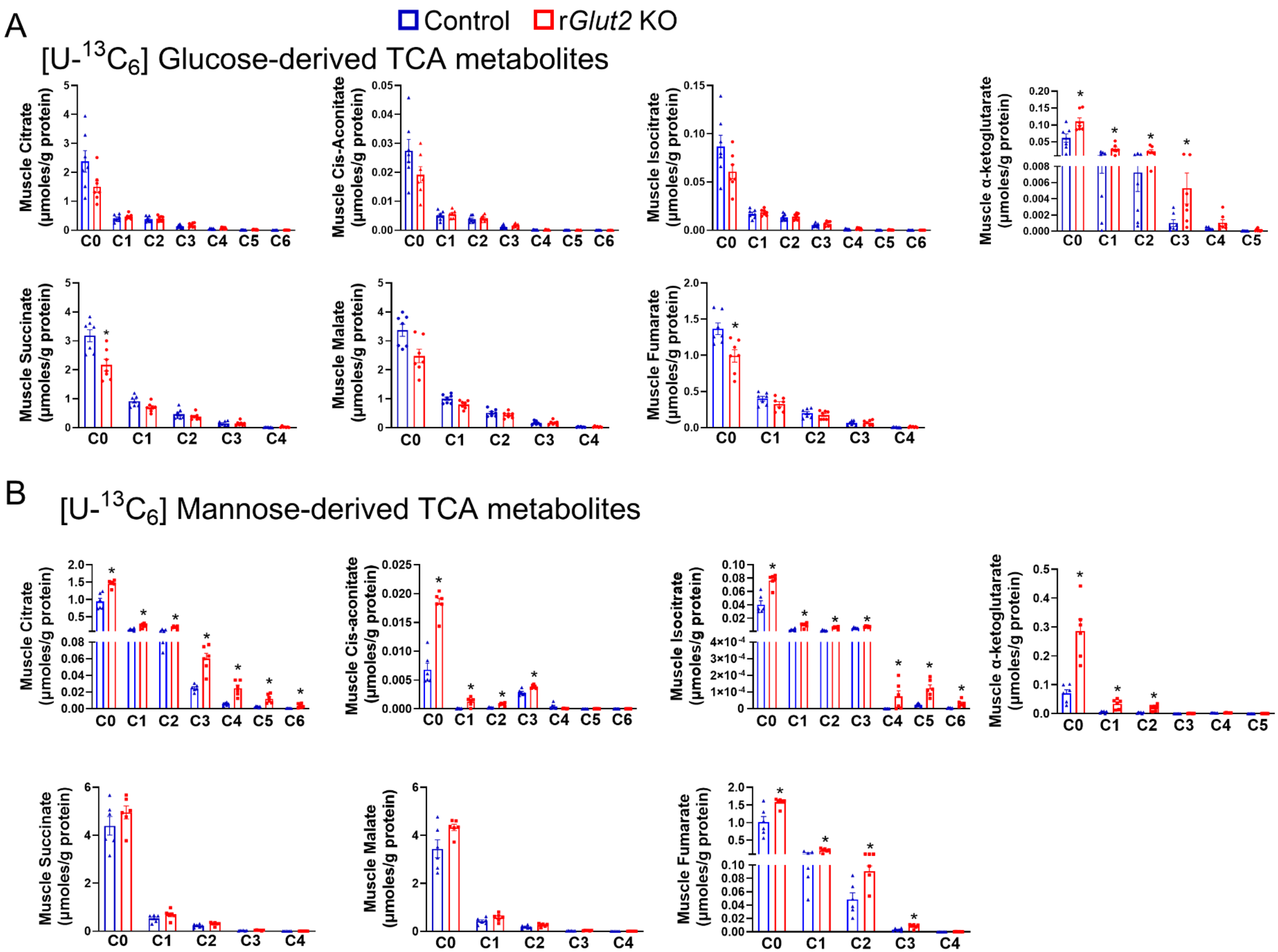

A
Control
rGlut2 KO
[U-13C6] Glucose-derived TCA metabolites
Muscle Citrate (µmoles/g protein)
Muscle Cis-Aconitate (µmoles/g protein)
Muscle Isocitrate (µmoles/g protein)
Muscle α-ketoglutarate (µmoles/g protein)
Muscle Succinate (µmoles/g protein)
Muscle Malate (µmoles/g protein)
Muscle Fumarate (µmoles/g protein)
C0
C1
C2
C3
C4
C5
C6
B
[U-13C6] Mannose-derived TCA metabolites
Muscle Citrate (µmoles/g protein)
Muscle Cis-aconitate (µmoles/g protein)
Muscle Isocitrate (µmoles/g protein)
Muscle α-ketoglutarate (µmoles/g protein)
Muscle Succinate (µmoles/g protein)
Muscle Malate (µmoles/g protein)
Muscle Fumarate (µmoles/g protein)

Figure S7. Stable isotope-resolved metabolomics analysis (by Ion Chromatography–Ultra-High Resolution Fourier Transform Mass Spectrometry) of soleus muscle TCA cycle metabolites in 14-week-old male r*Glut2* KO mice and their control littermates 60 minutes after $^{13}C_6$-glucose or $^{13}C_6$-mannose administration, related to Figure 5. **(A)** $^{13}C_6$-glucose labeling of TCA cycle intermediates (citrate, cis-aconitate, isocitrate, α-ketoglutarate, succinate, malate, and fumarate) was reduced or unchanged in rGlut2 KO mice, with the exception of α-ketoglutarate, which was increased, consistent with reduced contribution of glucose-derived carbon to TCA cycle intermediates in skeletal muscle. **(B)** $^{13}C_6$-mannose incorporation into TCA cycle intermediates (citrate, cis-aconitate, isocitrate, α-ketoglutarate, and fumarate) was increased in r*Glut2* KO mice (except succinate and malate, which were unchanged), consistent with increased contribution of mannose-derived carbon to TCA cycle intermediates in skeletal muscle. Data are shown as absolute metabolite abundance (μmol/g protein) for each isotopologue. The x-axis represents isotopologue distribution (C0–Cn), corresponding to the number of ^{13}C atoms incorporated into each metabolite. N=7, *p<0.05, multiple comparison unpaired t-test corrected using Holm-Sidak method. Data are represented as mean ± SEM.

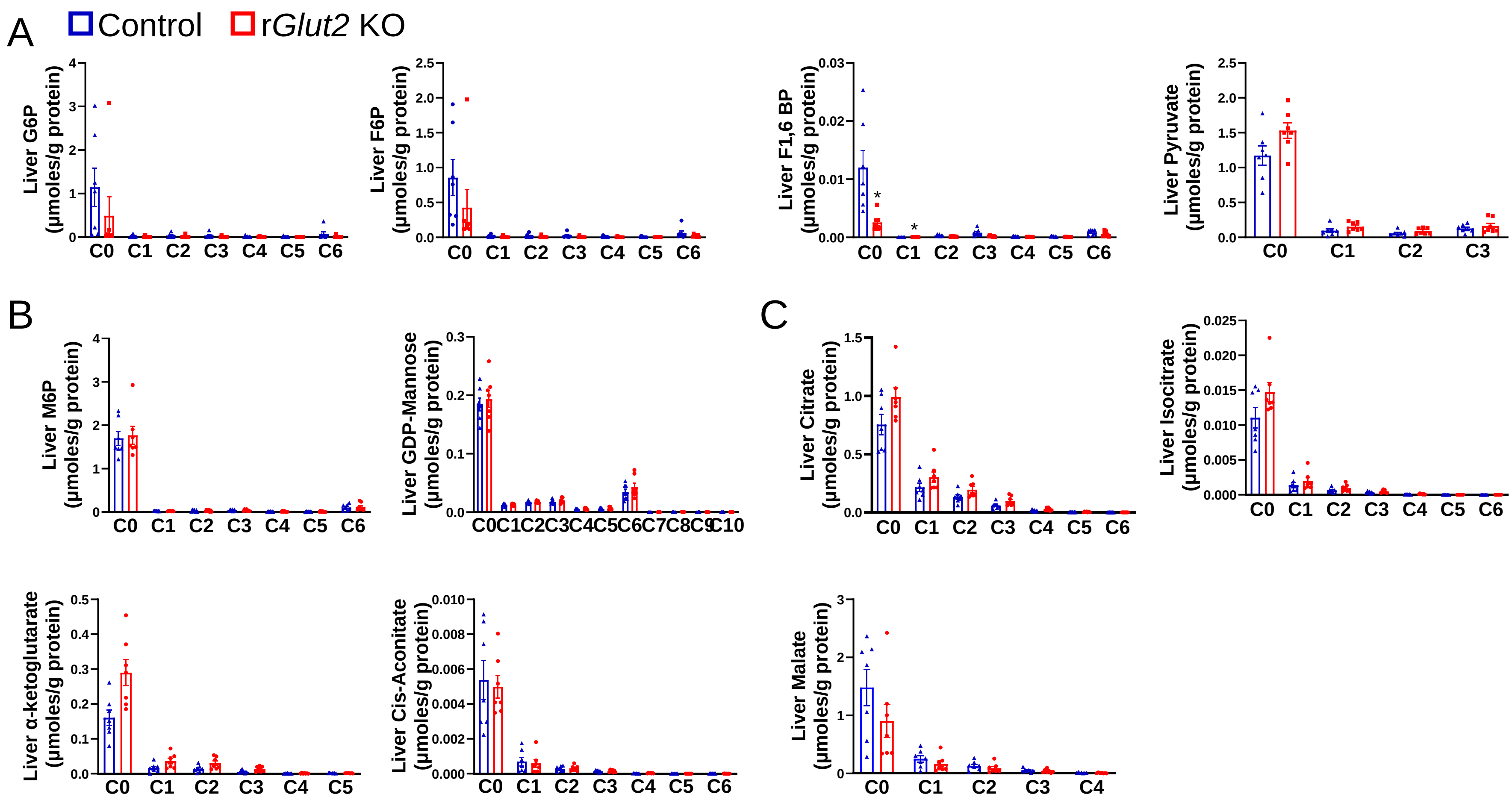

A
Control
r*Glut2* KO
Liver G6P (µmoles/g protein)
Liver F6P (µmoles/g protein)
Liver F1,6 BP (µmoles/g protein)
Liver Pyruvate (µmoles/g protein)
B
Liver M6P (µmoles/g protein)
Liver GDP-Mannose (µmoles/g protein)
C
Liver Citrate (µmoles/g protein)
Liver Isocitrate (µmoles/g protein)
Liver α-ketoglutarate (µmoles/g protein)
Liver Cis-Aconitate (µmoles/g protein)
Liver Malate (µmoles/g protein)
C0 C1 C2 C3 C4 C5 C6 C7 C8 C9 C10

Figure S8. Stable isotope-resolved metabolomics analysis (by Ion Chromatography–Ultra-High Resolution Fourier Transform Mass Spectrometry) of liver glucose and mannose metabolism in 14-week-old male r*Glut2* KO mice and their control littermates 60 minutes after $^{13}C_6$-glucose administration, related to Figures 1 and 2. **(A)** $^{13}C_6$-glucose incorporation into glucose-6-phosphate (G6P), fructose-6-phosphate (F6P), and pyruvate was similar between r*Glut2* KO mice and controls. For fructose-1,6-bisphosphate (F1,6BP), the unlabeled fraction (C0) was lower and C1 was higher in r*Glut2* KO liver, although the abundance of this intermediate was low in both groups. Overall, these data are consistent with no major differences in glycolytic or hexose-phosphate pathway labeling in the liver. **(B)** $^{13}C_6$-glucose incorporation into mannose pathway intermediates (mannose-6-phosphate [M6P] and GDP-mannose) was similar between groups, consistent with no major differences in mannose pathway labeling in liver. **(C)** $^{13}C_6$-glucose incorporation into TCA cycle intermediates was similar between r*Glut2* KO mice and controls, indicating no major differences in TCA cycle labeling in liver under these conditions. Data are shown as absolute metabolite abundance (µmol/g protein) for each isotopologue. The x-axis represents isotopologue distribution (C0–Cn), corresponding to the number of ^{13}C atoms incorporated into each metabolite. N=7, *$p<0.05$, multiple comparison unpaired t-test corrected using Holm-Sidak method. Data are represented as mean ± SEM.

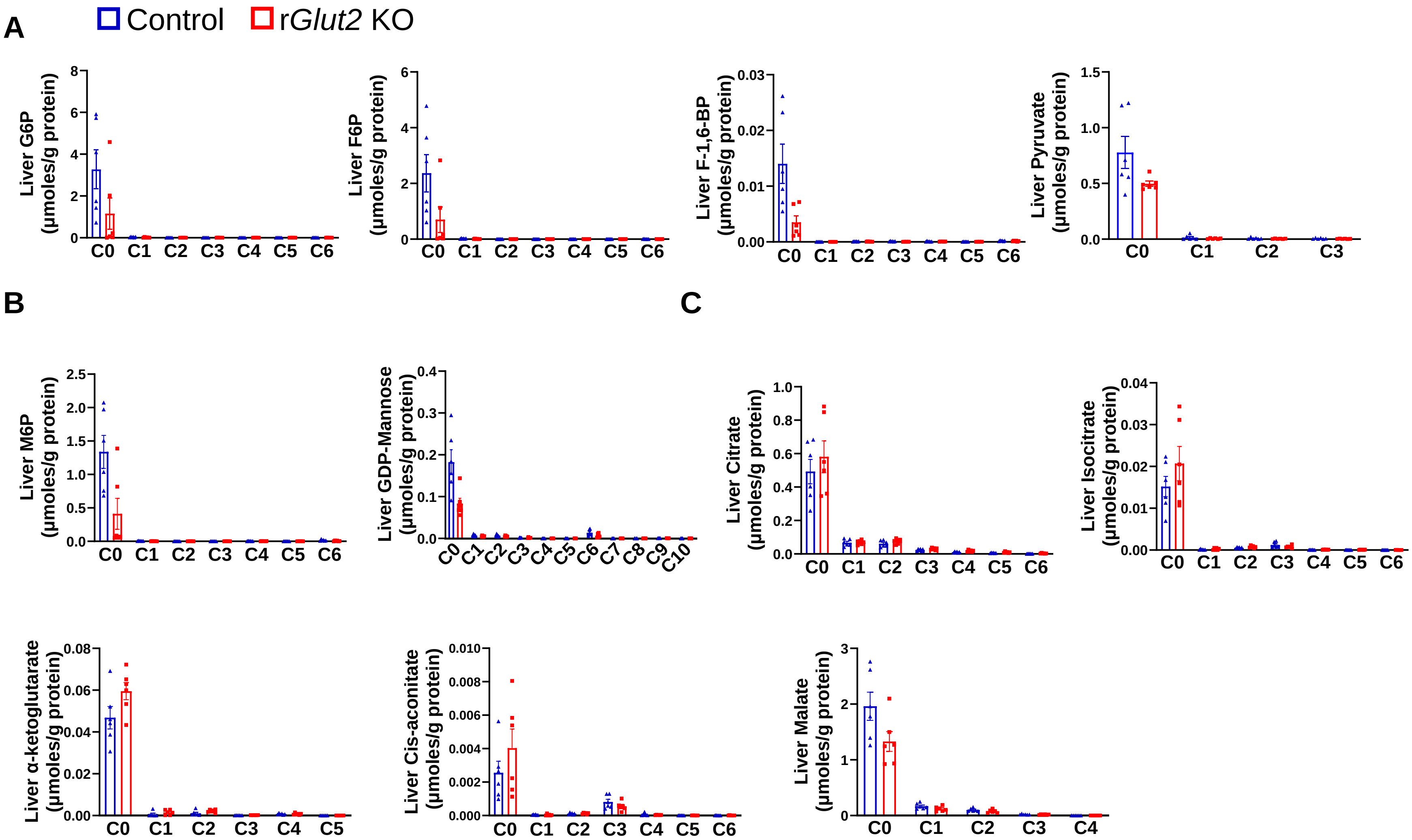

A
Control
rGlut2 KO
Liver G6P (µmoles/g protein)
Liver F6P (µmoles/g protein)
Liver F-1,6-BP (µmoles/g protein)
Liver Pyruvate (µmoles/g protein)
B
C
Liver M6P (µmoles/g protein)
Liver GDP-Mannose (µmoles/g protein)
Liver Citrate (µmoles/g protein)
Liver Isocitrate (µmoles/g protein)
Liver α-ketoglutarate (µmoles/g protein)
Liver Cis-aconitate (µmoles/g protein)
Liver Malate (µmoles/g protein)
C0
C1
C2
C3
C4
C5
C6
C7
C8
C9
C10

Figure S9. Stable isotope-resolved metabolomics analysis (by Ion Chromatography–Ultra-High Resolution Fourier Transform Mass Spectrometry) of liver glucose and mannose metabolism in 14-week-old male r*Glut2* KO mice and their control littermates 60 minutes after $^{13}C_6$-mannose administration, related to Figure 2. **(A)** $^{13}C_6$-mannose incorporation into glucose-6-phosphate (G6P), fructose-6-phosphate (F6P), fructose-1,6-bisphosphate (F1,6BP), and pyruvate was similar between r*Glut2* KO mice and controls, consistent with no major differences in hexose-phosphate or glycolytic pathway labeling. **(B)** $^{13}C_6$-mannose incorporation into mannose pathway intermediates (mannose-6-phosphate [M6P] and GDP-mannose) was similar between groups, consistent with no major differences in mannose pathway labeling in liver. **(C)** $^{13}C_6$-mannose incorporation into TCA cycle intermediates was similar between r*Glut2* KO mice and controls, consistent with no major differences in TCA cycle labeling in liver under these conditions. Data are shown as absolute metabolite abundance (μmol/g protein) for each isotopologue. The x-axis represents isotopologue distribution (C0–Cn), corresponding to the number of ^{13}C atoms incorporated into each metabolite. N=7. Data are represented as mean ± SEM.

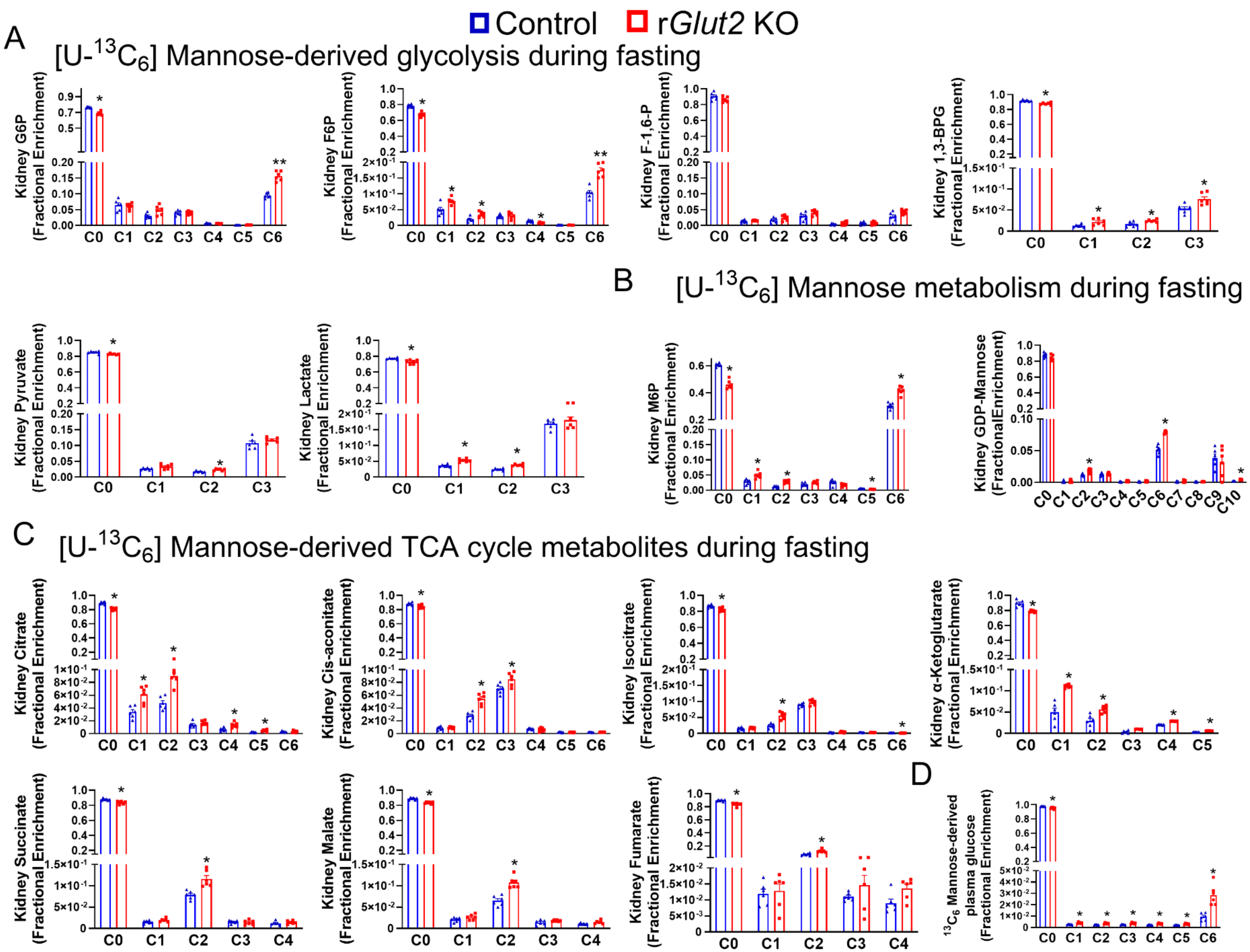

Control
rGlut2 KO
A
[U-13C6] Mannose-derived glycolysis during fasting
Kidney G6P (Fractional Enrichment)
Kidney F6P (Fractional Enrichment)
Kidney F-1,6-P (Fractional Enrichment)
Kidney 1,3-BPG (Fractional Enrichment)
Kidney Pyruvate (Fractional Enrichment)
Kidney Lactate (Fractional Enrichment)
B
[U-13C6] Mannose metabolism during fasting
Kidney M6P (Fractional Enrichment)
Kidney GDP-Mannose (FractionalEnrichment)
C
[U-13C6] Mannose-derived TCA cycle metabolites during fasting
Kidney Citrate (Fractional Enrichment)
Kidney Cis-aconitate (Fractional Enrichment)
Kidney Isocitrate (Fractional Enrichment)
Kidney α-Ketoglutarate (Fractional Enrichment)
Kidney Succinate (Fractional Enrichment)
Kidney Malate (Fractional Enrichment)
Kidney Fumarate (Fractional Enrichment)
D
13C6 Mannose-derived plasma glucose (Fractional Enrichment)
C0 C1 C2 C3 C4 C5 C6 C7 C8 C9 C10

Figure S10. Stable isotope-resolved metabolomics analysis (by Ion Chromatography–Ultra-High Resolution Fourier Transform Mass Spectrometry) of kidney glucose and mannose metabolism, including TCA cycle metabolites, in 14-week-old male r*Glut2* KO mice and their control littermates 60 minutes after oral $^{13}C_6$-mannose administration following a 6-hour fast, related to Figures 1 and 2. **(A)** $^{13}C_6$-mannose labeling of glycolytic intermediates glucose-6-phosphate (G6P), fructose-6-phosphate (F6P), fructose-1,6-bisphosphate (F1,6BP), pyruvate, 1,3-bisphosphoglycerate (1,3-BPG), and lactate. **(B)** $^{13}C_6$-mannose incorporation into mannose pathway intermediates (mannose-6-phosphate [M6P] and GDP-mannose). **(C)** $^{13}C_6$-mannose labeling of TCA cycle intermediates (citrate, cis-aconitate, isocitrate, α-ketoglutarate, succinate, malate, and fumarate). **(D)** $^{13}C_6$-mannose-derived labeling of plasma glucose, demonstrating incorporation of mannose-derived carbon into circulating glucose under fasting conditions, consistent with contribution of mannose to gluconeogenic pathways in vivo. Data are presented as fractional isotopologue enrichment. The x-axis represents isotopologue distribution (C0–Cn), corresponding to the number of ^{13}C atoms incorporated into each metabolite. N=7, *$p<0.05$, **$p<0.01$, multiple comparison unpaired t-test corrected using Holm-Sidak method. Data are represented as mean ± SEM.

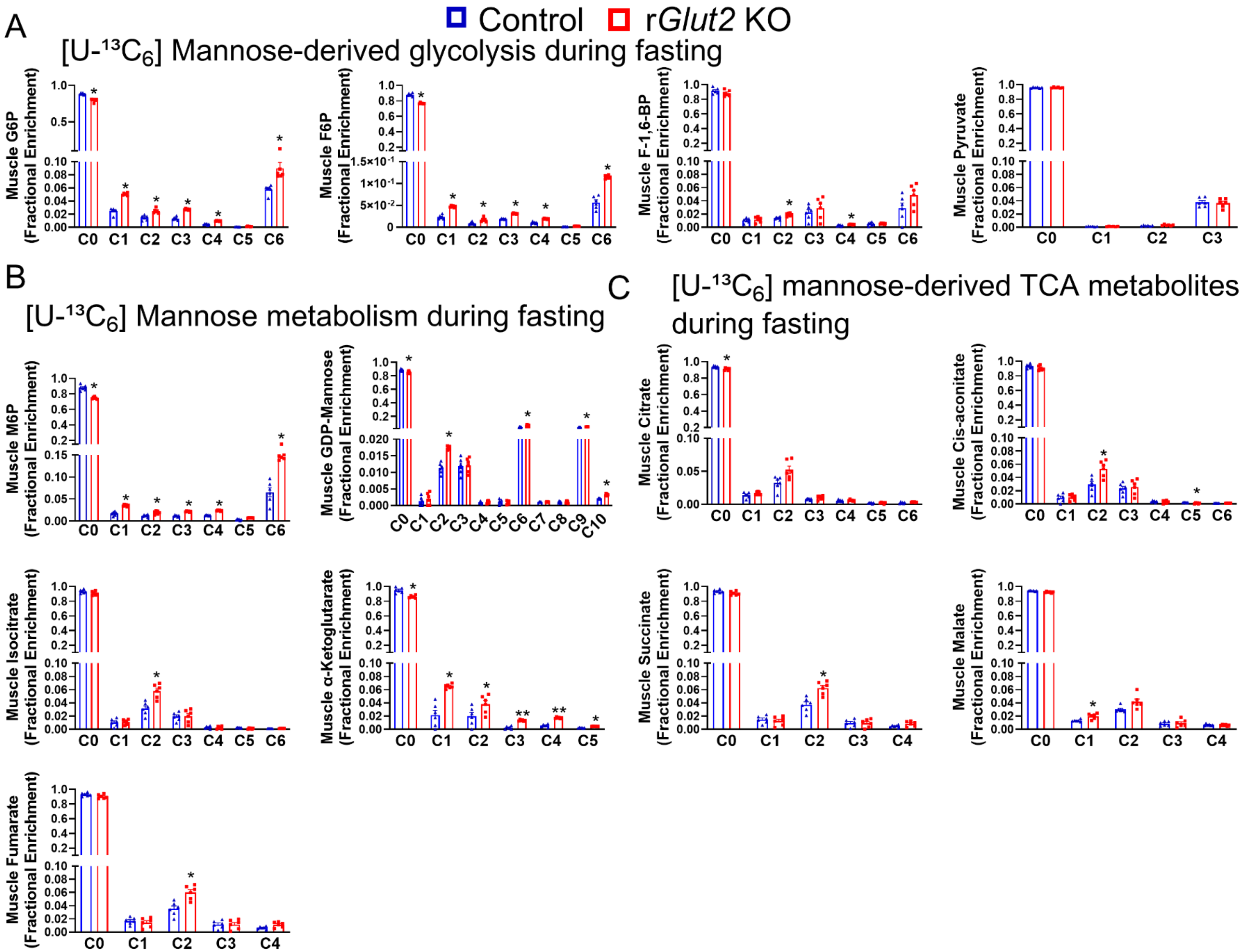
Control
rGlut2 KO
A
[U-13C6] Mannose-derived glycolysis during fasting
Muscle G6P (Fractional Enrichment)
Muscle F6P (Fractional Enrichment)
Muscle F-1,6-BP (Fractional Enrichment)
Muscle Pyruvate (Fractional Enrichment)
B
[U-13C6] Mannose metabolism during fasting
Muscle M6P (Fractional Enrichment)
Muscle GDP-Mannose (Fractional Enrichment)
Muscle Isocitrate (Fractional Enrichment)
Muscle α-Ketoglutarate (Fractional Enrichment)
Muscle Fumarate (Fractional Enrichment)
C
[U-13C6] mannose-derived TCA metabolites during fasting
Muscle Citrate (Fractional Enrichment)
Muscle Cis-aconitate (Fractional Enrichment)
Muscle Succinate (Fractional Enrichment)
Muscle Malate (Fractional Enrichment)

Figure S11. Stable isotope-resolved metabolomics analysis (by Ion Chromatography–Ultra-High Resolution Fourier Transform Mass Spectrometry) of soleus muscle glucose and mannose metabolism in 14-week-old male r*Glut2* KO mice and their control littermates 60 minutes after oral $^{13}C_6$-mannose administration following a 6-hour fast, related to Figures 1-5. **(A)** $^{13}C_6$-mannose labeling of glycolytic intermediates glucose-6-phosphate (G6P), fructose-6-phosphate (F6P), fructose-1,6-bisphosphate (F1,6BP), and pyruvate in soleus muscle. Labeling patterns indicated incorporation of mannose-derived carbon into glycolytic intermediates, with modest differences between r*Glut2* KO mice and controls. **(B)** $^{13}C_6$-mannose incorporation into mannose pathway intermediates (mannose-6-phosphate [M6P] and GDP-mannose) in soleus muscle. Incorporation was increased in r*Glut2* KO mice compared with controls, consistent with increased contribution of mannose-derived carbon to the hexose-phosphate pool. **(C)** $^{13}C_6$-mannose labeling of TCA cycle intermediates (citrate, cis-aconitate, isocitrate, α-ketoglutarate, succinate, malate, and fumarate) in soleus muscle. Labeling of several intermediates was increased in r*Glut2* KO mice, consistent with contribution of mannose-derived carbon to skeletal muscle oxidative metabolism under fasting conditions. Data are presented as fractional isotopologue enrichment. The x-axis represents isotopologue distribution (C0–Cn), corresponding to the number of ^{13}C atoms incorporated into each metabolite. N=7, *$p<0.05$, **$p<0.01$, multiple comparison unpaired t-test corrected using Holm-Sidak method. Data are represented as mean ± SEM.

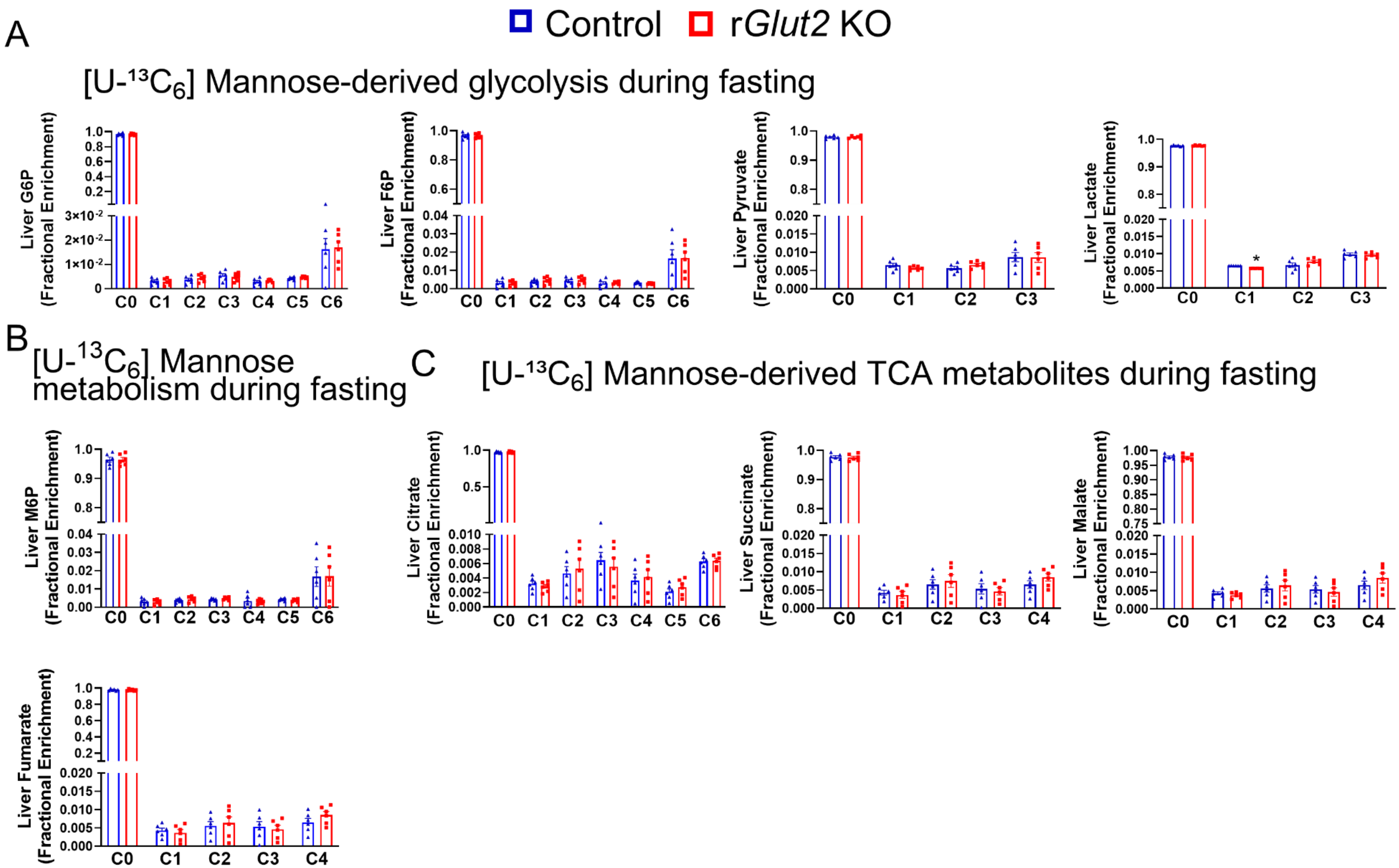

Control
rGlut2 KO
A
[U-13C6] Mannose-derived glycolysis during fasting
Liver G6P (Fractional Enrichment)
Liver F6P (Fractional Enrichment)
Liver Pyruvate (Fractional Enrichment)
Liver Lactate (Fractional Enrichment)
B
[U-13C6] Mannose metabolism during fasting
Liver M6P (Fractional Enrichment)
Liver Fumarate (Fractional Enrichment)
C
[U-13C6] Mannose-derived TCA metabolites during fasting
Liver Citrate (Fractional Enrichment)
Liver Succinate (Fractional Enrichment)
Liver Malate (Fractional Enrichment)
C0
C1
C2
C3
C4
C5
C6

Figure S12. Stable isotope-resolved metabolomics analysis (by Ion Chromatography–Ultra-High Resolution Fourier Transform Mass Spectrometry) of liver glucose and mannose metabolism, including TCA cycle metabolites, in 14-week-old male r*Glut2* KO mice and their control littermates 60 minutes after oral $^{13}C_6$-mannose administration following a 6-hour fast, related to Figures 1-5. **(A)** $^{13}C_6$-mannose labeling of glycolytic intermediates glucose-6-phosphate (G6P), fructose-6-phosphate (F6P), pyruvate, and lactate in the liver. Labeling patterns were broadly similar between r*Glut2* KO mice and controls, consistent with no major differences in glycolytic or hexose-phosphate pathway labeling under fasting conditions. **(B)** $^{13}C_6$-mannose incorporation into mannose pathway intermediate, mannose-6-phosphate [M6P], in the liver. Incorporation patterns were similar between groups, consistent with no major differences in mannose pathway labeling. **(C)** $^{13}C_6$-mannose labeling of TCA cycle intermediates (citrate, succinate, malate, and fumarate) in the liver. Labeling was comparable between r*Glut2* KO mice and controls, consistent with no major differences in TCA cycle labeling under these conditions. Data are presented as fractional isotopologue enrichment. The x-axis represents isotopologue distribution (C0–Cn), corresponding to the number of ^{13}C atoms incorporated into each metabolite. N=7, *$p<0.05$, multiple comparison unpaired t-test corrected using Holm-Sidak method. Data are represented as mean ± SEM.

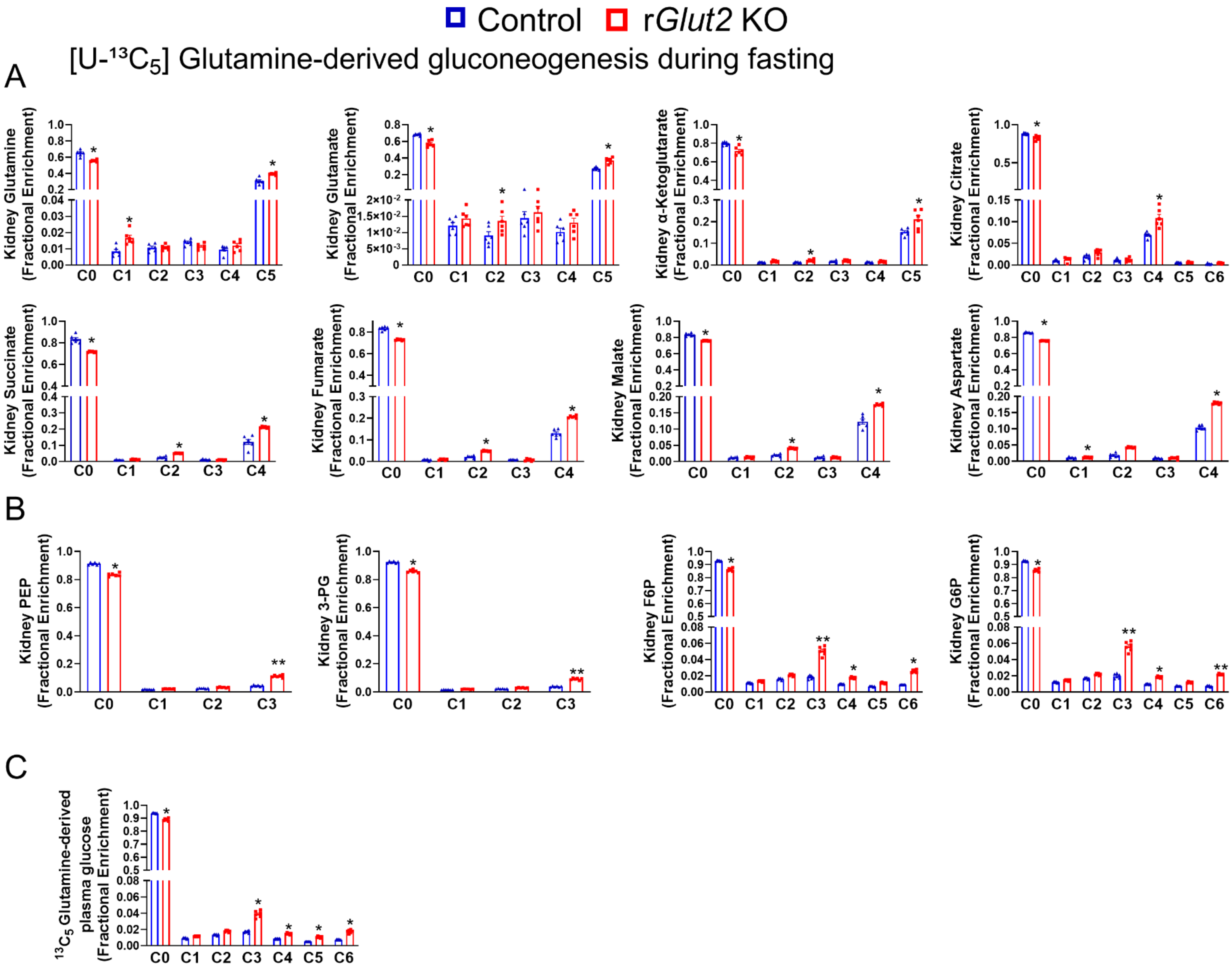
Control
rGlut2 KO
[U-13C5] Glutamine-derived gluconeogenesis during fasting
A
Kidney Glutamine (Fractional Enrichment)
Kidney Glutamate (Fractional Enrichment)
Kidney α-Ketoglutarate (Fractional Enrichment)
Kidney Citrate (Fractional Enrichment)
Kidney Succinate (Fractional Enrichment)
Kidney Fumarate (Fractional Enrichment)
Kidney Malate (Fractional Enrichment)
Kidney Aspartate (Fractional Enrichment)
B
Kidney PEP (Fractional Enrichment)
Kidney 3-PG (Fractional Enrichment)
Kidney F6P (Fractional Enrichment)
Kidney G6P (Fractional Enrichment)
C
13C5 Glutamine-derived plasma glucose (Fractional Enrichment)
C0 C1 C2 C3 C4 C5 C6

Figure S13. Stable isotope-resolved metabolomics analysis (by Ion Chromatography–Ultra-High Resolution Fourier Transform Mass Spectrometry) of kidney glutamine metabolism in 14-week-old male r*Glut2* KO mice and their control littermates 60 minutes after intraperitoneal $^{13}C_5$-glutamine administration following a 6-hour fast, related to Figures 1-3. **(A)** $^{13}C_5$-glutamine incorporation into TCA cycle entry metabolites, including glutamine, glutamate, α-ketoglutarate, and citrate, in r*Glut2* KO and control kidneys. Labeling of these intermediates was increased in r*Glut2* KO mice, consistent with enhanced glutamine-derived anaplerotic input into the TCA cycle. Within the same panel, labeling of downstream TCA cycle intermediates (succinate, fumarate, malate, and aspartate) was also increased in r*Glut2* KO mice, consistent with propagation of glutamine-derived carbon through the TCA cycle. **(B)** $^{13}C_5$-glutamine incorporation into gluconeogenic intermediates phosphoenolpyruvate (PEP), 3-phosphoglycerate (3-PG), fructose-6-phosphate (F6P), and glucose-6-phosphate (G6P). **(C)** $^{13}C_5$-glutamine-derived labeling of plasma glucose. Labeling of these intermediates and of plasma glucose was increased in r*Glut2* KO mice, consistent with contribution of glutamine-derived carbon to gluconeogenic pathways *in vivo*. Data are presented as fractional isotopologue enrichment. The x-axis represents isotopologue distribution (C0–Cn), corresponding to the number of ^{13}C atoms incorporated into each metabolite. N=7, *$p<0.05$, **$p<0.01$, multiple comparison unpaired t-test corrected using Holm-Sidak method. Data are represented as mean ± SEM.

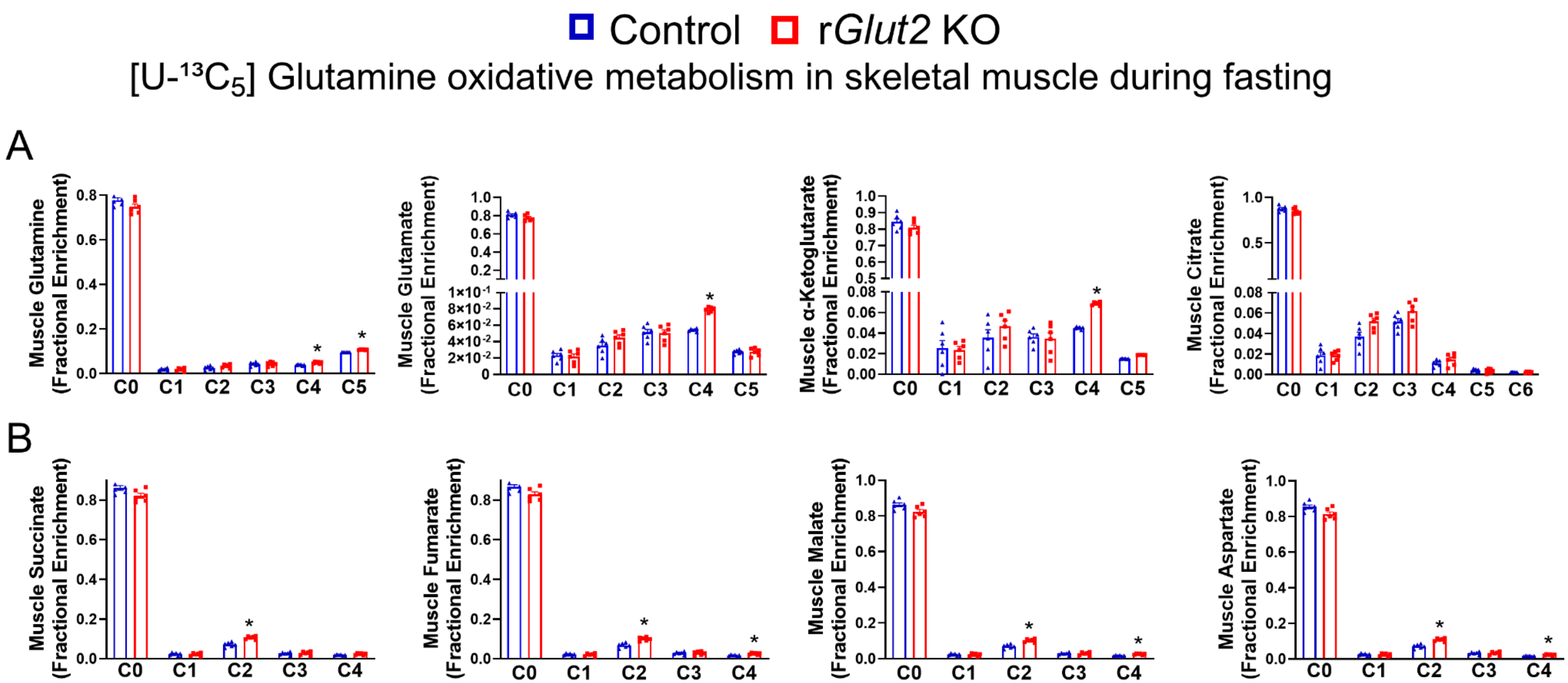
Control
rGlut2 KO
[U-13C5] Glutamine oxidative metabolism in skeletal muscle during fasting
A
Muscle Glutamine (Fractional Enrichment)
Muscle Glutamate (Fractional Enrichment)
Muscle α-Ketoglutarate (Fractional Enrichment)
Muscle Citrate (Fractional Enrichment)
B
Muscle Succinate (Fractional Enrichment)
Muscle Fumarate (Fractional Enrichment)
Muscle Malate (Fractional Enrichment)
Muscle Aspartate (Fractional Enrichment)
C0
C1
C2
C3
C4
C5
C6

Figure S14. Stable isotope-resolved metabolomics analysis (by Ion Chromatography–Ultra-High Resolution Fourier Transform Mass Spectrometry) of soleus muscle glutamine metabolism in 14-week-old male r*Glut2* KO mice and their control littermates 60 minutes after intraperitoneal $^{13}C_5$-glutamine administration following a 6-hour fast, related to Figure 5. **(A)** $^{13}C_5$-glutamine incorporation into TCA cycle entry metabolites (glutamine, glutamate, α-ketoglutarate, and citrate) in soleus muscle. Labeling of glutamine, glutamate, and α-ketoglutarate was modestly increased in r*Glut2* KO mice compared with controls, whereas citrate was unchanged, consistent with increased contribution of glutamine-derived carbon to TCA cycle entry. **(B)** $^{13}C_5$-glutamine labeling of downstream TCA cycle intermediates, including succinate, fumarate, malate, and aspartate in soleus muscle. Labeling of these metabolites was modestly increased or similar between groups, consistent with limited propagation of glutamine-derived carbon through the TCA cycle under fasting conditions. Data are presented as fractional isotopologue enrichment. The x-axis represents isotopologue distribution (C0–Cn), corresponding to the number of ^{13}C atoms incorporated into each metabolite. N=7, *$p<0.05$, multiple comparison unpaired t-test corrected using Holm-Sidak method. Data are represented as mean ± SEM.

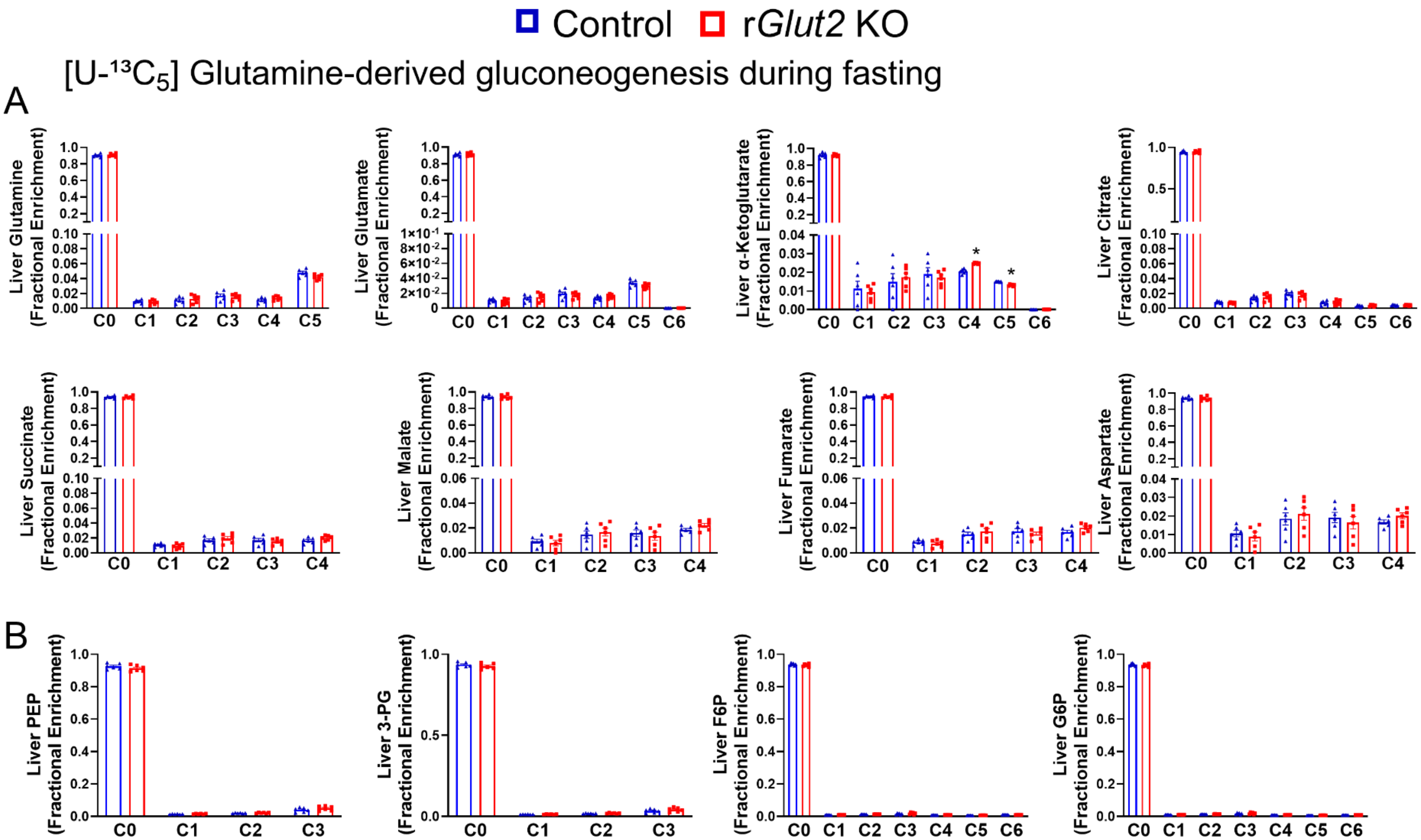

Control
rGlut2 KO
[U-13C5] Glutamine-derived gluconeogenesis during fasting
A
Liver Glutamine (Fractional Enrichment)
C0 C1 C2 C3 C4 C5
Liver Glutamate (Fractional Enrichment)
C0 C1 C2 C3 C4 C5 C6
Liver α-Ketoglutarate (Fractional Enrichment)
C0 C1 C2 C3 C4 C5 C6
Liver Citrate (Fractional Enrichment)
C0 C1 C2 C3 C4 C5 C6
Liver Succinate (Fractional Enrichment)
C0 C1 C2 C3 C4
Liver Malate (Fractional Enrichment)
C0 C1 C2 C3 C4
Liver Fumarate (Fractional Enrichment)
C0 C1 C2 C3 C4
Liver Aspartate (Fractional Enrichment)
C0 C1 C2 C3 C4
B
Liver PEP (Fractional Enrichment)
C0 C1 C2 C3
Liver 3-PG (Fractional Enrichment)
C0 C1 C2 C3
Liver F6P (Fractional Enrichment)
C0 C1 C2 C3 C4 C5 C6
Liver G6P (Fractional Enrichment)
C0 C1 C2 C3 C4 C5 C6

Figure S15. Stable isotope-resolved metabolomics analysis (by Ion Chromatography–Ultra-High Resolution Fourier Transform Mass Spectrometry) of liver glutamine metabolism in 14-week-old male r*Glut2* KO mice and their control littermates 60 minutes after intraperitoneal $^{13}C_5$-glutamine administration following a 6-hour fast, related to Figures 1-5. **(A)** $^{13}C_5$-glutamine incorporation into TCA cycle intermediates, including glutamine, glutamate, α-ketoglutarate, citrate, succinate, malate, fumarate, and aspartate in the liver. Labeling patterns were similar between r*Glut2* KO mice and controls, consistent with no major differences in glutamine-derived TCA cycle metabolism. **(B)** $^{13}C_5$-glutamine incorporation into gluconeogenic intermediates phosphoenolpyruvate (PEP), 3-phosphoglycerate (3-PG), fructose-6-phosphate (F6P), and glucose-6-phosphate (G6P) in the liver. Labeling of these intermediates was low and comparable between groups, consistent with limited contribution of glutamine-derived carbon to gluconeogenic pathways under these conditions. Data are presented as fractional isotopologue enrichment. The x-axis represents isotopologue distribution (C0–Cn), corresponding to the number of ^{13}C atoms incorporated into each metabolite. N=7, *$p<0.05$, multiple comparison unpaired t-test corrected using Holm-Sidak method. Data are represented as mean ± SEM.

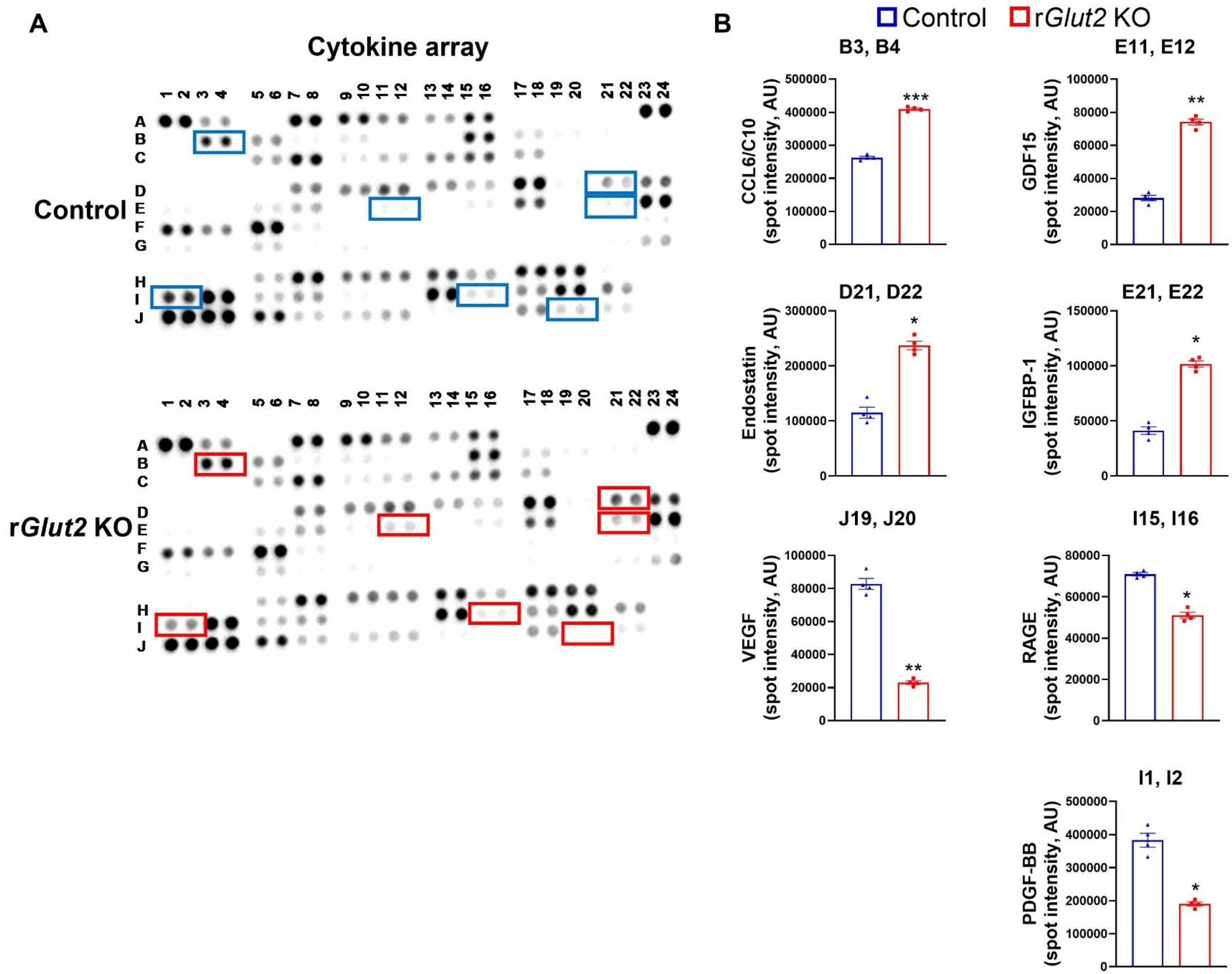

A
Cytokine array
Control
rGlut2 KO
B
Control
rGlut2 KO
B3, B4
CCL6/C10 (spot intensity, AU)
E11, E12
GDF15 (spot intensity, AU)
D21, D22
Endostatin (spot intensity, AU)
E21, E22
IGFBP-1 (spot intensity, AU)
J19, J20
VEGF (spot intensity, AU)
I15, I16
RAGE (spot intensity, AU)
I1, I2
PDGF-BB (spot intensity, AU)

Figure S16. Cytokine array analysis of serum from 12-week-old male r*Glut2* KO mice and their control littermates, related to Figure 7. **(A)** Representative cytokine array dot blot of serum samples from control and r*Glut2* KO mice. **(B)** Densitometric quantification of selected cytokines showing significant differences between groups. The coordinates indicated in each graph title correspond to the position of the immobilized antibody for each cytokine on the array shown in **(A)**. Several cytokines were increased (CCL6/C10, GDF15, Endostatin, and IGFBP-1), whereas others were decreased (VEGF, RAGE, and PDGF-BB) in r*Glut2* KO mice, consistent with systemic alterations in circulating cytokines associated with renal glycosuria. N=4 pooled samples per group; 50 µl serum from three mice was pooled to generate one sample, resulting in four pooled samples from 12 mice per group. Statistical significance was determined by two-tailed unpaired t-test (*$p<0.05$, **$p<0.01$, ***$p<0.001$). CCL6/C10, chemokine (C-C motif) ligand 6; GDF15, growth differentiation factor 15; IGFBP-1, insulin-like growth factor binding protein 1; VEGF, vascular endothelial growth factor; RAGE, receptor for advanced glycation end-products; PDGF-BB, platelet-derived growth factor-BB. Data are presented as exploratory due to the pooled serum design; individual-level variability cannot be inferred. Data are represented as mean ± SEM.
